# Systems biology-defined NF-*κ*B regulons, interacting signal pathways and networks are implicated in the malignant phenotype of head and neck cancer cell lines differing in p53 status

**DOI:** 10.1186/gb-2008-9-3-r53

**Published:** 2008-03-11

**Authors:** Bin Yan, Guang Chen, Kunal Saigal, Xinping Yang, Shane T Jensen, Carter Van Waes, Christian J Stoeckert, Zhong Chen

**Affiliations:** 1 Head and Neck Surgery Branch, NIDCD, National Institutes of Health, Bethesda, MD 20892, USA; 2 Department of Bioengineering, Smith Walk; University of Pennsylvania, Philadelphia, Pennsylvania 19104, USA; 3 Center for Bioinformatics, Guardian Drive; University of Pennsylvania, Philadelphia, Pennsylvania 19104, USA; 4 NIH-Pfizer Clinical Research Training Program Award; University of Pennsylvania, Philadelphia, Pennsylvania 19104, USA; 5 Department of Statistics, The Wharton School, Walnut Street; University of Pennsylvania, Philadelphia, Pennsylvania 19104, USA; 6 Department of Genetics, School of Medicine, Curie Boulevard; University of Pennsylvania, Philadelphia, Pennsylvania 19104, USA

## Abstract

**Background:**

Aberrant activation of the nuclear factor kappaB (NF-*κ*B) pathway has been previously implicated as a crucial signal promoting tumorigenesis. However, how NF-*κ*B acts as a key regulatory node to modulate global gene expression, and contributes to the malignant heterogeneity of head and neck cancer, is not well understood.

**Results:**

To address this question, we used a newly developed computational strategy, COGRIM (Clustering Of Gene Regulons using Integrated Modeling), to identify NF-*κ*B regulons (a set of genes under regulation of the same transcription factor) for 1,265 genes differentially expressed by head and neck cancer cell lines differing in p53 status. There were 748 NF-*κ*B targets predicted and individually annotated for RELA, NF*κ*B1 or cREL regulation, and a prevalence of RELA related genes was observed in over-expressed clusters in a tumor subset. Using Ingenuity Pathway Analysis, the NF-*κ*B targets were reverse-engineered into annotated signature networks and pathways, revealing relationships broadly altered in cancer lines (activated proinflammatory and down-regulated Wnt/β-catenin and transforming growth factor-*β *pathways), or specifically defective in cancer subsets (growth factors, cytokines, integrins, receptors and intermediate kinases). Representatives of predicted NF-*κ*B target genes were experimentally validated through modulation by tumor necrosis factor-*α *or small interfering RNA for *RELA *or *NFκB1*.

**Conclusion:**

NF-*κ*B globally regulates diverse gene programs that are organized in signal networks and pathways differing in cancer subsets with distinct p53 status. The concerted alterations in gene expression patterns reflect cross-talk among NF-*κ*B and other pathways, which may provide a basis for molecular classifications and targeted therapeutics for heterogeneous subsets of head and neck or other cancers.

## Abstract 2

Detailed analysis of NF*κ*B regulons in 1,265 genes differentially expressed in head and neck cancer cell lines differing in p53 status revealed a cross talk between NFkB and specific signaling pathways.

## Background

The nuclear factor kappaB (NF-*κ*B) family comprises a group of evolutionarily conserved signal-activated transcription factors (TFs) that have been shown to play a central role in the control of a large number of normal and stressed cellular processes [1,2]. NF-*κ*B is involved in similar biological processes in cancers, as a critical modulator of genes that promote cell survival, inflammation, angiogenesis, tumor development, progression and metastasis [3-5]. We previously showed that NF-*κ*B is aberrantly activated and modulates the expression of gene clusters that include oncogenes that promote survival, tumorigenesis and therapeutic resistance of advanced murine and human squamous cell carcinomas [6-16]. In addition, NF-*κ*B and related pathways have been identified as potential biomarkers and therapeutic targets for a variety of human cancers [3,4,17-19]. However, our understanding of the regulatory mechanisms activating or affected by the NF-*κ*B pathway still remains limited to the classical concept of linear pathway activation based on experimental observations from traditional biological approaches. Such a linear paradigm for NF-*κ*B as well as other pathways could be problematic, as suggested by the observation that pharmacological and clinical approaches targeting individual NF-*κ*B signal molecules alone have not yielded significant clinical efficacy in most solid tumors [20-22].

Several levels of complexity contribute to our limited understanding of the function of the NF-*κ*B pathway in health and disease. First, the NF-*κ*B family consists of five structurally related proteins, namely RELA (p65), NF*κ*B1 (p50/p105), cREL, RELB, and NF*κ*B2 (p52/p100), as well as seven inhibitor kappaB (I*κ*B) molecules [1,2]. Constitutive activation of RELA/NF*κ*B1 was found to be an essential factor controlling the expression of genes that affect cellular proliferation, apoptosis, angiogenesis, immune and proinflammatory responses, and therapeutic resistance in head and neck squamous cell carcinoma (HNSCC) and other cancers [3-5]. However, nuclear activation of hetero- and homodimers composed of other NF-*κ*B subunits has also been detected in HNSCC tissues and cell lines [23]. While the function of the less studied species of NF-*κ*B is not yet fully understood, there is evidence that formation of homo- or heterodimers from different NF-*κ*B subunits can increase the diversity of responses through interaction with various I*κ*Bs or other regulatory factors, and by having different binding affinities for variant *κ*B promoter binding motifs [1,2,24]. Second, multiple signals from membrane receptors and intermediate kinases converge to modulate different NF-*κ*B subunits directly or indirectly. At present, there is evidence for signaling through a classic pathway involving a trimeric inhibitor-kappaB kinase (IKK)*α*/*β*/*γ *and casein kinase 2 complexes modulating NF*κ*B1, RELA and cREL, and alternative pathways involving NF-*κ*B inducing kinase and IKK*α *modulating NF*κ*B2 and RELB [1,2,11,24-26]. Furthermore, there is potential for cross-talk between IKK/NF-*κ*B and other major signal pathways, such as the mitogen-activated protein kinase (MAPK), phosphatidylinositol 3-kinase (PI3K), JAK/STAT (Janus kinase/signal transducer and transcription factor), and p53 pathways, which have been implicated in significantly affecting the cancer phenotype, including proliferation, apoptosis, angiogenesis and tumorigenesis [1,4,27-30]. These observations highlight the tremendous technical challenges and experimental limitations when studying such dynamic and complex biological and regulatory systems using a classic one molecule/one pathway approach.

Molecular and phenotypic heterogeneity represents an additional obstacle that limits our understanding of the regulatory mechanisms giving rise to differences in the malignant phenotype between different cancers of the same histological type, such as HNSCC. The identification of heterogeneous sub-populations in specific types of cancer, such as HNSCC, and selection of therapies targeting them are major hurdles for clinical diagnosis, prognosis and treatment. Such heterogeneity usually remains undetected by standard histological and pathological classification and clinical grading systems, and other biomarkers based on molecular gene expression profiles and immunohistochemistry are not yet well enough understood or validated for clinical applications. Such heterogeneity in the malignant phenotype includes differences in prognosis, therapeutic resistance, angiogenesis or metastatic potential associated with specific molecular alterations identified in HNSCC, such as overexpression or mutation of epidermal growth factor receptor (EGFR) [10,31,32], constitutive activation of NF-*κ*B, MAPK, AKT and STAT pathways [15,31,33-37], mutation or dysfunction of p53/p63/p73 family members [35,36,38], and over-expression of proinflammatory and proangiogeneic cytokines and growth factors, including interleukin (IL)1, IL6, IL8, vascular endothelial growth factor (VEGF), platelet-derived growth factor, and hepatocyte growth factor [18,34,37,39-42].

We recently identified specific gene expression signatures in HNSCC cell lines (UM-SCC, University of Michigan Cell Lines Series of Head and Neck Squamous Cell Carcinoma), which were associated with differing p53 status and NF-*κ*B regulatory activity, subsets previously associated with differences in prognosis, response to chemoradiation or metastatic phenotypes [14]. Some genes in the NF-*κ*B related expression signatures identified from our study have been identified and associated with a higher risk for HNSCC recurrence and metastasis by independent groups [43,44]. However, the individual genes and proteins identified from the molecular and clinical studies do not function alone, but often form dynamically complex interactions to execute their biological functions, through regulatory control mechanisms involving TFs, signal pathways and networks. The analysis of critical transcriptional modules, pathways and networks has been experimentally impractical, until the recent availability of large sets of data from different microarray and genomic platforms, as well as advances in development of bioinformatic and systems biology approaches [45,46].

It remains a great challenge to systematically analyze transcriptional regulation in eukaryotes through mathematical modeling and integration of multiple large data sets from different platforms and experimental conditions, where each provides only partial information about the biological process. To address these challenges, a statistical model, COGRIM (Clustering of Gene Regulons Using Integrated Modeling) has been developed, based on a Bayesian hierarchical model with a Markov chain Monte Carlo implementation [47,48]. Here, this modeling has been specifically applied to novel applications in human cancer cell lines, where the successful prediction of NF-*κ*B regulons (a set of genes under regulation of the same TF) in HNSCC cell lines has been achieved by integration of large data sets of gene expression and multiple TFs from different platforms and experimental conditions. Furthermore, the global connections of NF-*κ*B regulons were established through networks and pathways using Ingenuity Pathway Analysis (IPA), and predicted novel NF-*κ*B targets were confirmed with experimental validation. Our study identified distinct molecular signatures composed of NF-*κ*B dominant signal pathways and networks specific for subsets of HNSCC cell lines differing in p53 status. Our identification of NF-*κ*B related networks and pathways could significantly enhance our understanding of NF-*κ*B regulatory mechanisms, lead to new concepts of molecular regulation and classification of cancer subgroups, and targeted therapeutics for HNSCC.

## Results

### Genome-wide identification of NF-*κ*B target genes in HNSCC cell lines through COGRIM modeling

**Figure 1:**
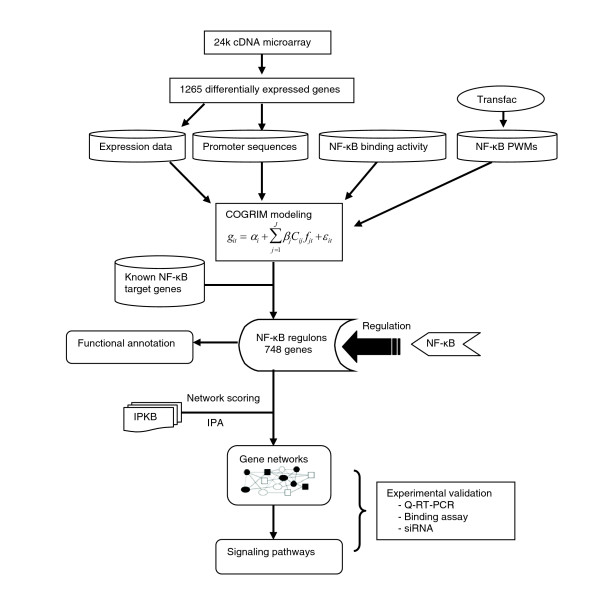
A schematic diagram of computational, analytic and experimental strategies. COGRIM modeling was performed by integrating four data sources, including microarray analysis of genes differentially expressed by cancer cells, the promoter sequences extracted from genomic databases, NF-*κ*B binding activity in cancer cells, and the NF-*κ*B PWMs from Transfac. The predicted NF-*κ*B target genes were subjected to Ingenuity Pathway Analysis, and NF-*κ*B-associated networks and signaling pathways were identified. The predicted NF-*κ*B target genes were validated by real time RT-PCR, gene knocking down by siRNA, and NF-*κ*B specific binding assays.

Previously, heterogeneous gene expression signatures were identified in the UM-SCC cell lines associated with different p53 status [14]. In this study, NF-*κ*B target genes were predicted by COGRIM modeling from 1,265 genes differentially expressed in UM-SCC cells, and subgrouped by their p53 status (Figure 1). A total of 748 genes were identified as putative NF-*κ*B target genes, which represented 59% of the differentially expressed genes input (Figure 1 and Additional data file 1). Among the 748 genes, 10% (75 genes) were previously identified as NF-*κ*B target genes (labeled in bold in Additional data file 1), based on publications from PubMed and available web sites described in the Materials and methods section. These known NF-*κ*B target genes, such as *IL6*, *IL8*, *BIRC2 (clAP-1)*, *ICAM1*, *YAP1*, *CDKN1A *(p21), *CSF2*, *CCDN1*, *IL1A*, *IL1B*, and so on, include many that have been independently confirmed to be differentially expressed and pathologically implicated in HNSCC and other cancers [6-8,39,44,49-52]. In addition, functional binding of activated NF-*κ*B to several sites within the promoters of *IL6*, *IL8*, *ICAM1 *and *YAP1 *have been confirmed experimentally in our laboratory [6,14].

**Figure 2:**
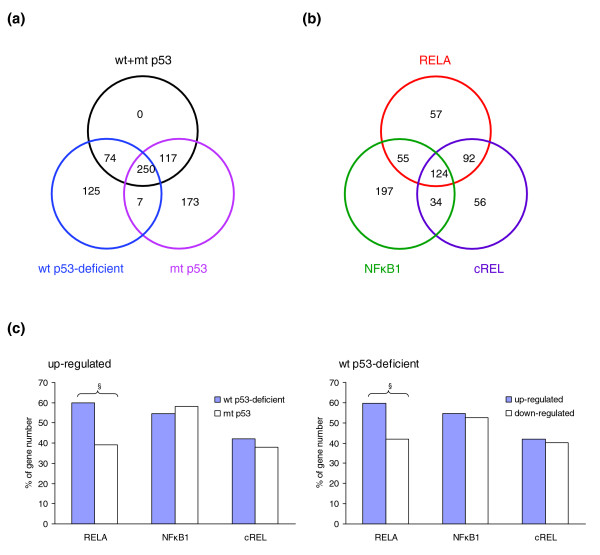
Distribution of predicted NF-*κ*B target genes. **(a) **The distribution of predicted NF-*κ*B target genes in UM-SCC cells with different p53 status using five NF-*κ*B binding PWMs. **(b) **The distribution of predicted genes regulated by RELA, NF*κ*B1, or cREL using individual PWMs. **(c) **Comparison of distribution (%) of predicted genes by RELA, NF*κ*B1, or cREL regulation in the up-regulated gene group of UM-SCC cells (left), and in the cells with wild-type p53-deficient status (right). ^§^Statistical significance by chi square (*X*^2^, *P *< 0.001).

Next, we investigated if differentially expressed NF-*κ*B target genes were specifically associated with subgroups of UM-SCC cell lines that differ in p53 status (Figure 2a). Among these NF-*κ*B target genes, 125 were associated with wild-type (wt) p53-deficient status [14], 173 were associated with mutant (mt) p53 status, and 250 were globally expressed in UM-SCC cells (wt+mt p53) relative to non-malignant keratinocytes (Figure 2a). In addition, 74 genes were overlapping between the group of lines with wild-type p53-deficient status and all 10 p53 cell lines used (wt+mt), which include the 5 cell lines with wild-type p53-deficient status. Similarly, 117 genes were overlapping between the group of 5 cell lines with mutant p53 status and the 10 wt+mt p53 cell lines. Seven genes overlapped among cell groups with either wild-type or mutant p53 status, which are mutually exclusive groups; however, these seven genes showed either up- or down-regulation in the different groups of cells, indicating that they could be oppositely affected by p53 status. Furthermore, we annotated specific genes under regulation by three individual NF-*κ*B subunits, RELA, NF*κ*B1 or cREL. There were 124 genes predicted to be under the regulation of all three NF-*κ*B subunits; 328 genes by RELA; 410 genes by NF*κ*B1; and 306 genes by cREL (Figure 2b and Additional data file 1). In addition, some genes were predicted to be preferentially under the regulation of one of the NF-*κ*B family members, including 57 genes under RELA regulation, 197 genes under NF*κ*B1 regulation, and 56 genes under cREL regulation (Figure 2b). We also observed that genes preferentially under RELA regulation were over-represented in the up-regulated genes in the subgroup of tumors with wild-type p53-deficient status (*Χ*^2 ^analysis, *P *< 0.0001; Figure 2c). Thus, our study predicted broad associations between NF-*κ*B regulated genes with all UM-SCC groups, or with subsets of them that differ in p53 status, and, specifically, it revealed an over-representation of RELA up-regulated genes in UM-SCC cell lines with wild-type p53-deficient status.

### Predicted functionality of putative NF-*κ*B target genes by comparative genomics

The identification of conserved NF-*κ*B binding sites across human and mouse genomes was conducted through a comparative genome analysis (Transfac 8.4), as these binding sites are more likely to be evolutionarily important and functional. We observed that 183 of 748 genes (24.5%) have conserved NF-*κ*B binding sites, including *IL6*, *ICAM1*, *REL(cREL)*, *TIMP2*, *CSF1*, *IL1A*, *IL1B*, *IL1R2*, *ITGA5*, *LAMB3*, and so on (Additional data file 1). Individually, conserved RELA, NF*κ*B1 or cREL binding sites were identified in the promoters of 73 (22.3%), 96 (23.4%) and 67 (21.9%) genes, respectively (Additional data file 1). To determine the functional classification of the NF-*κ*B target genes, we performed Gene Ontology annotation. Among the top Gene Ontology categories, epidermal development, cell differentiation, angiogenesis, cell-cell signaling, and cell adhesion appeared in all tumor groups with increased statistical significance (Additional data file 2).

### NF-*κ*B regulon related networks

**Figure 3:**
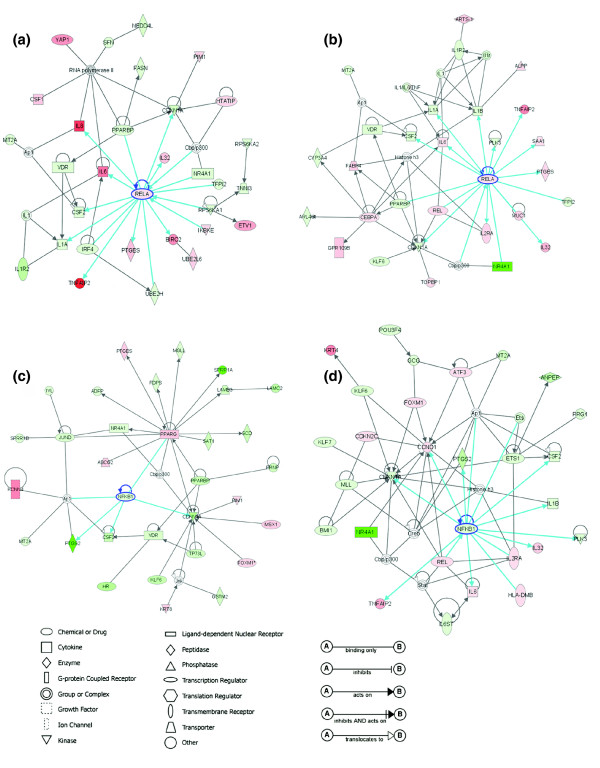
RELA or NF*κ*B1 dominant networks revealed by IPA. **(a, b) **RELA or **(c, d) **NF*κ*B1 dominant networks in cells with wild-type p53-deficient (a, c) or mutant p53 (b, d) status were generated by IPA and showed graphically. The brightness of node colors is proportional to the fold changes of gene expression levels. Color indicates up-regulated (red) and down-regulated (green) genes. Blue lines indicate direct connections of RELA or NF*κ*B1 with genes through different functionalities.

It has been hypothesized that NF-*κ*B promotes cancer cell progression through interactions with other proteins, associated signal pathways and structured biological networks [1,2,4,26]. Using COGRIM modeling, we predicted NF-*κ*B regulons, which refer to the sets of genes under regulation of specific TFs, such as NF-*κ*B RELA. Using IPA, we examined how NF-*κ*B regulons connected as networks in cells with different p53 status. IPA defines networks as a group of biologically related genes, proteins or other molecules based on experimentally derived genomic datasets and relationships through dynamical computation and manual extraction of thousands of direct and indirect physical and functional interactions from peer-reviewed publications. The relationships in the network include protein-protein interactions, protein binding to DNA or RNA, protein enzyme and substrate interactions, as well as transcriptional and translational regulation, as described in Figure 3.

We observed that RELA or NF*κ*B1 dominant networks ranked top in each subset of cells (Figure 3 and Additional data file 3), consistent with the importance of NF-*κ*B regulons predicted by COGRIM. Specifically, in cells with wild-type p53-deficient status, the top-ranked network with *RELA *included: seven up-regulated genes (compared with human normal keratinocytes), such as *IL6*, *IL8*, *BIRC2*, *TNFAIP2*, *IKBKE*, and so on; nine down-regulated genes, such as *IL1A*, *CSF2*, *CDKN1A*, and so on; plus four molecular complexes/groups, such as cAMP responsive element binding protein and p300 (CBP/p300), IL1, activating protein-1 (AP1) and RNA polymerase II (Figure 3a). In cells with mutant p53 status, the top-ranked network with *RELA *included: seven up-regulated genes, such as *IL6*, *REL*, *IL2RA*, *TNFAIP2*, and so on; eight down-regulated genes, such as *IL1A*, *IL1B*, *CSF2*, *CDKN1A*, and so on; plus several complexes/groups, such as CBP/p300, AP1, IL1/IL6/tumor necrosis factor (TNF), IL1 receptor (IL1R) and histone H3 (Figure 3b). In the top-ranked network related to *NFκB1*, only four genes were identified in cells with wild-type p53-deficient status: *PPARG*, *CDKN1A*, *CSF2*, *PTGS2*, plus AP1 complex (Figure 3c). In cells with mutant p53 status, NF*κ*B1 was linked with seven up-regulated genes, such as *CCDN1*, *IL6*, *REL*, *TNFAIP2*, and so on; five down-regulated genes, such as *CDKN1A*, *ETS1*, *CSF2*, and so on; plus six complexes/groups, such as CBP/p300, AP1, CREB (cAMP Responsive Element Binding Protein), STAT, ETS and histone H3 (Figure 3d). Here we noticed that there were exceptionally fewer NF*κ*B1 target genes connected in cells with wild-type p53-deficient status. Thus, the network analyses revealed potentially unique interactive relationships of NF-*κ*B regulons in the subgroups of cells with different p53 status.

### NF-*κ*B regulon associated signal pathways

**Figure 4:**
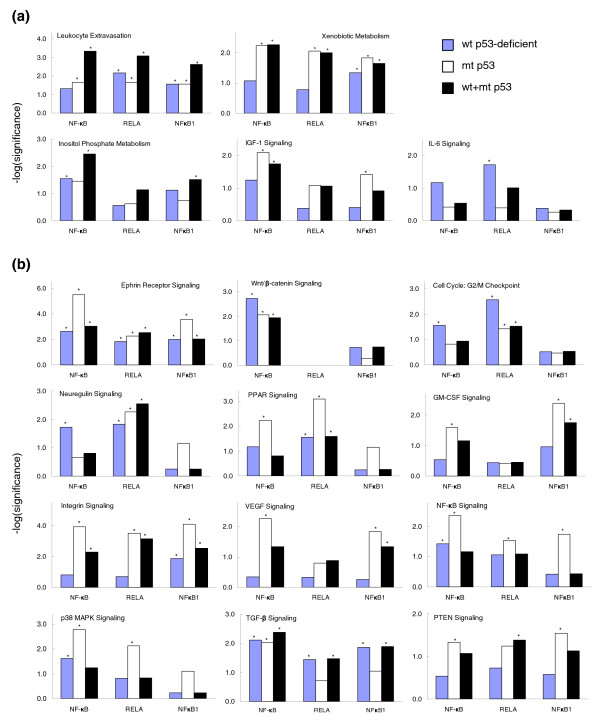
NF-*κ*B target genes were reverse-engineered and assigned to signaling pathways with significant implication in the malignant phenotype. NF-*κ*B target genes were analyzed by IPA and the pathways with statistical significance were presented. The y-axis represents the statistical significances in log scale of each signaling pathway, and the x-axis indicates the predicted genes specifically regulated by NF-*κ*B subunits. On the x-axis, 'NF-*κ*B' refers to common NF-*κ*B regulation (not subunit specific), and 'RELA' and 'NF*κ*B1' refer to regulation by RELA or NF*κ*B1 subunits, respectively. **(a) **Pathways associated with up-regulated genes in cancer cells with different p53 statuses; **(b) **pathways associated with down-regulated genes. *Pathways that reached a statistically significant level (*P *< 0.05).

Next, we analyzed how NF-*κ*B regulons are related to other signal pathways using IPA with a significance level of *P *< 0.05; relationships to different NF-*κ*B subunits, such as RELA and NF*κ*B1, were determined and are shown in Figure 4. A detailed list of genes involved in each pathway is presented in Table 1. Figure 4a shows, for the pathways composed of the up-regulated genes in the broader panel of UM-SCC cells, that all NF-*κ*B family members were associated with the pathways of leukocyte extravasation, inositol phosphate metabolism and xenobiotic metabolism (top panels and left panel in the second row). Insulin-like growth factor (IGF) signaling was significantly associated with all NF-*κ*B family members in tumor cells with mutant p53 status (middle panel in the second row). However, genes involved in the IL-6 signaling pathway were most significantly associated with RELA in cells with wild-type p53 status (right panel of the second row). When the genes down-regulated broadly in UM-SCC cells were analyzed (Figure 4b), Wnt/*β*-catenin signaling and transforming growth factor (TGF)-*β *signaling pathways were related to all NF-*κ*B family members, while RELA was dominantly associated with components of the neuregulin signaling pathway (the third row). In the remaining signaling and functional pathways, with the exception of cell cycle:G2/M checkpoint components, different NF-*κ*B subunits were associated with down-regulated genes in cells with mutant p53 status, whereas cell cycle:G2/M checkpoint was the only pathway associated more significantly with RELA in cells with wild-type p53-deficient status (Figure 4b, rows 4-6). The analysis provides evidence for potential differences in the contribution of NF-*κ*B subunits in the regulation of genes involved in the signature pathways of the subset tumor cells with different p53 status.

**Table 1:** Signal pathways associated with NF-*κ*B regulons in UM-SCC cells

Tumor type*	Pathway	p53^†^	*P*-value^‡^	Genes^§^
All subgroups	Ephrin receptor signaling	W	8.1 × 10^-3^	*ANGPT1*↓, *CXCL14*↓, *EFNB1*↓, *EPHB2*↑, *EPHB4*↓, *ITGA2*↓, *GNA15*↓, *GNAI2*↓, *GNB1*↓, *GNG12*↓, *IL8*↑, *PGF*↓
		M	2.3 × 10^-3^	*AKT1*↓, *ANGPT1*↓, *AXIN1*↑, *CXCL14*↑, *EFNB1*↓, *GNA15*↓, *GNAI2*↓, *GNB2*↓, *GNB4*↓, *GNG12*↓, *ITGA2*↓, *MAP4K4*↓, *PGF*↓, *RASA1*↑, *RAC2*↓, *RHOA*↓, *VEGFC*↓
		W+M	8.9 × 10^-4^	*ANGPT1*↓, *AXIN1*↑, *CXCL14*↑, *EFNB1*↓, *EPHB2*↑, *GNAI2*↓, *GNA15*↓, *GNG12*↓, *IL8*↑, *ITGA2*↓, *PGF*↓, *RAC2*↓, *RHOA*↓, *VEGFC*↓
	Leukocyte extravasation signaling	W	4.4 × 10^-2^	*CD99*↓, *CLDN7*↑, *CXCL14*↓, *CYBA*↑, *GNAI2*↓, *ICAM1*↑, *IL8*↑, *PRKCQ*↓, *TIMP2*↑, *VASP*↓
		M	1.8 × 10^-3^	*ACTN3*↓, *ACTG2*↓, *CD99*↓, *CD44*↓, *CLDN7*↑, *CXCL14*↑, *CYBA*↑, *GNAI2*↓, *MMP13*↑, *PIK3R3*↑, *PLCG2*↑, *RAC2*↓, *RHOA*↓, *TIMP2*↑, *VASP*↓
		W+M	7.9 × 10^-5^	*ACTN3*↓, *CD99*↓, *CLDN7*↑, *CXCL14*↑, *CYBA*↑, *GNAI2*↓, *ICAM1*↑, *IL8*↑, *MMP13*↑, *PIK3R3*↑, *PLCG2*↑, *PRKCQ*↓, *RAC2*↓, *RHOA*↓, *TIMP2*↑, *VASP*↓
	Wnt/β-catenin signaling	W	3.2 × 10^-2^	*DKK3*↓, *GJA1*↓, *PPP2R5B*↓, *SFRP1*↓, *SOX8*↓, *SOX9*↓, *TCF4*↓, *TGFBR2*↓, *TLE4*↓
		M	3.4 × 10^-2^	*AKT1*↓, *AXIN1*↑, *CCND1*↑, *CD44*↓, *DKK3*↓, *SOX9*↓, *SFRP1*↓, *TCF4*↓, *TGFB2*↓, *TGFBR2*↓
		W+M	2.8 × 10^-2^	*AXIN1*↑, *CCND1*↑, *DKK3*↓, *PPP2R5B*↓, *SFRP1*↓, *SOX8*↓, *SOX9*↓, *TCF4*↓ *TGFBR2*↓
	Xenobiotic metabolism signaling	W	1.2 × 10^-2^	*ALDH1A3*↑, *ALDH4A1*↓, *ALDH5A1*↑, *FMO3*↓, *GSTM2*↓, *IL1A*↓, *IL6*↑, *NOS2A*↓, *NQO1*↑, *PPARBP*↓, *PPP2R5B*↓, *PRKCQ*↓, *SULT1A3*↑
		M	8.7 × 10^-3^	*ALDH1A2*↑, *ALDH1A3*↑, *ALDH3B2*↑, *CYP1A2*↑, *CYP3A4*↓, *EIF2AK3*↓, *FMO3*↓, *IL1A*↓, *IL1B*↓, *IL6*↑, *NFE2L2*↑, *NQO1*↑, *PIK3R3*↑, *PPARBP*↓, *SULT1A3*↑
		W+M	1.6 × 10^-3^	*ALDH1A2*↑, *ALDH5A1*↑, *ALDH1A3*↑, *ALDH3B2*↑, *CYP3A4*↓, *FMO3*↓, *IL1A*↓, *IL6*↑, *NOS2A*↓, *NQO1*↑, *PIK3R3*↑, *PPARBP*↓, *PPP2R5B*↓, *PRKCQ*↓, *SULT1A3*↑
	ERK/MAPK signaling	W+M	4.2 × 10^-2^	*DUSP4*↓, *DUSP6*↓, *ELF3*↑, *ETS1*↓, *ITGA2*↓, *PIK3R3*↑, *PLCG2*↑, *PPP2R5B*↓, *PPARG*↑, *RAC2*↓
	Inositol phosphate metabolism	W+M	1.7 × 10^-2^	*ISYNA1*↑, *ITPKA*↑, *NEK2*↑, *PIK3R3*↑, *PIM1*↑, *PLK1*↑, *PRKCQ*↓, *PLCD1*↓, *PLCG2*↑, *PRKX*↓
	IL-6 signaling	W	4.4 × 10^-2^	*IKBKE*↑, *IL1A*↓, *IL1R2*↓, *IL1RN*↓, *IL6*↑, *IL8*↑
		M	1.7 × 10^-2^	*IL1A*↓, *IL1B*↓, *IL1R2*↓, *IL6*↑, *IL6ST*↓, *TNFRSF1A*↓, *MAP4K4*↓, *LBP*↑
	p38 MAPK signaling	W	4.8 × 10^-2^	*DUSP10*↑, *IL1A*↓, *IL1R2*↓, *IL1RN*↓, *MAPKAPK3*↓, *TGFBR2*↓
		M	3.5 × 10^-3^	*DUSP10*↑, *IL1A*↓, *IL1B*↓, *IL1R2*↓, *MAPKAPK3*↓, *PLA2G4B*↑, *TGFB2*↓, *TGFBR2*↓, *TNFRSF1A*↓
Wild-type p53-deficient	Cell cycle:G2/M DNA damage	W	3.5 × 10^-3^	*CDKN1A*↓, *PLK1*↑, *RPS6KA1*↓, *SFN*↓, *TOP2A*↑
	checkpoint regulation	W+M	1.8 × 10^-2^	*CDKN1A*↓, *PLK1*↑, *SFN*↓, *TOP2A*↑
	Neuregulin signaling	W	3.4 × 10^-2^	*ADAM17*↓, *ITGA2*↓, *NRG2*↓, *PDK1*↑, *PICK1*↓, *PRKCQ*↓
	PPAR signaling	W	3.6 × 10^-2^	*IL1A*↓, *IL1R2*↓, *IL1RN*↓, *IKBKE*↑, *PPARBP*↓, *PPARG*↑
	Protein ubiquitination pathway	W	3.1 × 10^-2^	*BIRC2*↑, *CDC20*↑, *DOC1*↓, *FBXW7*↓, *NEDD4L*↓, *PSMB10*↑, *SMURF2*↓, *UBE2H*↓, *UBE2L6*↑, *USP6*↓
Mutant p53	GM-CSF signaling	M	1.5 × 10^-2^	*AKT1*↓, *CCND1*↑, *CFS2*↓, *ETS1*↓, *PIK3R3*↑, *PPP3CC*↓
		W+M	6.0 × 10^-3^	*CCND1*↑, *CFS2*↓, *ETS1*↓, *PIK3R3*↑, *PIM1*↑, *PPP3CC*↓
	IGF-1 signaling	M	2.0 × 10^-3^	*AKT1*↓, *CYR61*↓, *IGFBP2*↑, *IGFBP3*↑, *IGFBP6*↑, *IRS1*↑, *PIK3R3*↑, *RASA1*↑, *SFN*↓
		W+M	3.0 × 10^-2^	*CYR61*↓, *IGFBP2*↑, *IGFBP3*↑, *IGFBP6*↑, *PIK3R3*↑, *SFN*↓
	Integrin signaling	M	1.3 × 10^-3^	*ACTG2*↓, *ACTN3*↓, *AKT1*↓, *BCAR3*↓, *DDEF1*↓, *ITGA2*↓, *ITGA5*↓, *ITGB4*↓, *LAMA3*↓, *LAMB3*↓, *LAMC2*↓, *PIK3R3*↑, *PLCG2*↑, *RAC2*↓, *RHOA*↓, *RHOC*↓, *TSPAN4*↓, *TSPAN7*↑, *VASP*↓
		W+M	2.6 × 10^-2^	*ACTN3*↓, *ITGA2*↓, *ITGA5*↓, *ITGA6*↓, *ITGB4*↓, *LAMA3*↓, *LAMB3*↓, *LAMC2*↓, *PIK3R3*↑, *PLCG2*↑, *RAC2*↓, *RHOA*↓, *RHOC*↓, *VASP*↓
	VEGF signaling	M	7.8 × 10^-3^	*ACTG2*↓, *ACTN3*↓, *AKT1*↓, *PGF*↓, *PIK3R3*↑, *PLCG2*↑, *SFN*↓, *VEGFC*↓
		W+M	3.1 × 10^-2^	*ACTN3*↓, *PGF*↓, *PIK3R3*↑, *PLCG2*↑, *SFN*↓, *VEGFC*↓
	NF-*κ*B signaling	M	1.7 × 10^-2^	*AKT1*↓, *BCL10*↓, *IL1A*↓, *IL1R2*↓, *IL1B*↓, *MALT1*↓, *MAP4K4*↓, *PIK3R3*↑, *PLCG2*↑, *TNFRSF1A*↓
	SAPK/JNK signaling	M	2.0 × 10^-2^	*DUSP4*↓, *DUSP10*↑, *EDG5*↓, *IRS1*↑, *MAP4K4*↓, *PIK3R3*↑, *RAC2*↓, *SH2D2A*↓, *ZAK*↓

Shown are signaling pathways associated with NF-*κ*B regulons in UM-SCC cells using IPA 5.0 with a significant enrichment (*P *< 0.05). *Subgroups with different p53 statuses that are associated with the major signal transduction pathways. ^†^The subgroups within each pathway based on p53 status: W refers to five UM-SCC cell lines with wild-type-deficient status; M refers to five UM-SCC cell lines with mutant p53 status; and W+M refers to ten UM-SCC cell lines. ^‡^Statistical significance of a given pathway (cut off, *P *< 0.05). ^§^Genes included in the pathway by IPA; up and down arrows indicate up- and down-regulated gene expression with two-fold or more changes.

### Modulation of NF-*κ*B target gene expression by TNF-*α *and small interfering RNA

**Figure 5:**
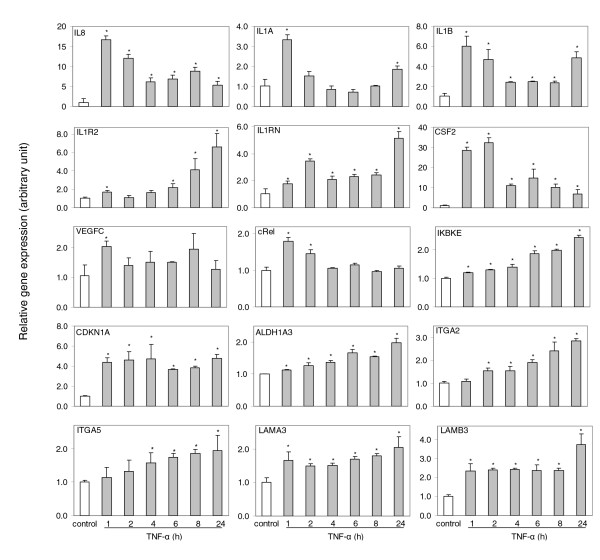
Basal and inducible expression of NF-*κ*B target genes modulated by TNF-*α*. UM-SCC 6 cells were treated with TNF-*α *(2000 units/ml) for different times. Total RNA was isolated, and genes selected from NF-*κ*B networks or pathways were analyzed by real time RT-PCR. The data are presented as the mean plus standard deviation from triplicates with normalization by 18S ribosome RNA. *P < 0.05 compared with the control (*t *test).

The predicted NF-*κ*B target genes involved in the networks and pathways were first validated by experimental modulation of gene expression under TNF-*α*, a classic NF-*κ*B inducer. We previously showed that TNF-*α *regulated a wide set of genes from one of the over-expressed clusters in UM-SCC, including *AKAP12*, *BAG2*, *ICAM1*, *IGFBP3*, *IL6*, *IL8*, *TNFAIP2*, and *PIK3R3 *[14]. In this study, we tested another 14 genes identified in NF-*κ*B related networks and pathways, including *IL8 *as a positive control (Figure 5). Expression of the genes modulated by TNF-*α *showed different kinetics. This included one group consisting of *IL8*, *IL1A*, *IL1B*, *CSF2*, *REL*, and *VEGFC*, which showed a rapid induction pattern typical of early response genes, where the peak of gene induction was observed around 1-2 hours with a rapid tapering back to the base line. In contrast, gene expression of *IL1R2*, *IKBKE*, *ALDH1A3*, *ITGA2 *and *ITGA5 *exhibited a slower time dependent induction (Figure 5).

**Figure 6:**
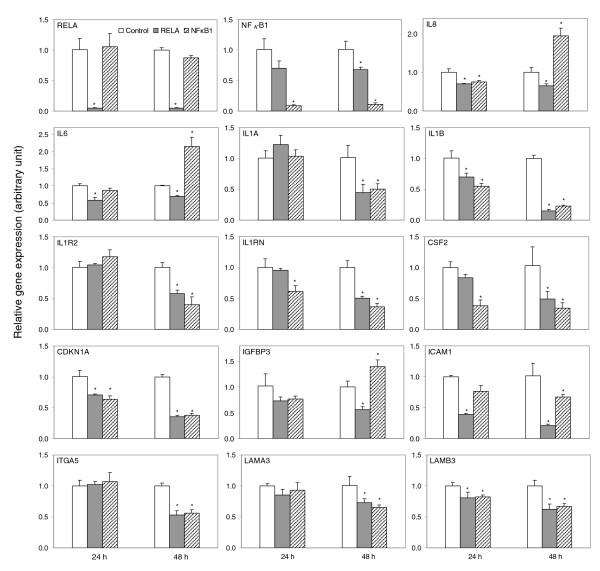
Silencing *RELA *or *NFκB1 *by siRNA significantly altered gene expression. UM-SCC 6 cells were transfected with siRNA to *RELA *or *NFκB1 *for 24 or 48 hours. Total RNA was isolated, and genes selected from NF-*κ*B networks or pathways were analyzed by real time RT-PCR. The data were calculated as the mean plus standard deviation from triplicates with normalization by 18S ribosome RNA, and are presented as the comparison with the cultured cells transfected with the control siRNA oligos. *P < 0.05 (*t *test).

To further examine whether the expression of predicted NF-*κ*B target genes was affected by NF-*κ*B subunits RELA or NF*κ*B1, we knocked down *RELA *or *NFκB1 *individually by small interfering RNAs (siRNAs). As shown in Figure 6, after knocking down *RELA *or *NFκB1 *for 24 or 48 hours, the expression levels of *RELA *or *NFκB1 *were dramatically reduced by more than 90% compared with control siRNA. Knocking down *RELA *reduced *NFκB1 *gene expression significantly at 48 hours and slightly decreased *IL8*, *IL6 *and *IGFBP3 *expression. However, knocking down *NFκB1 *significantly increased the gene expression at 48 hours, suggesting that NF*κ*B1 may mediate suppression of basal expression of these genes. Furthermore, knocking down *RELA *or *NFκB1 *suppressed *IL1A*, *IL1B*, *IL1R2*, *IL1RN*, *CSF2*, *CDKN1A*, *ITGA5*, *LAMA3 *and *LAMB3 *genes, more significantly at 48 hours. The expression of *ICAM1 *was affected more significantly by knocking down *RELA *than *NFκB1*.

### The binding activities of RELA and NF*κ*B1 in UM-SCC cells

**Figure 7:**
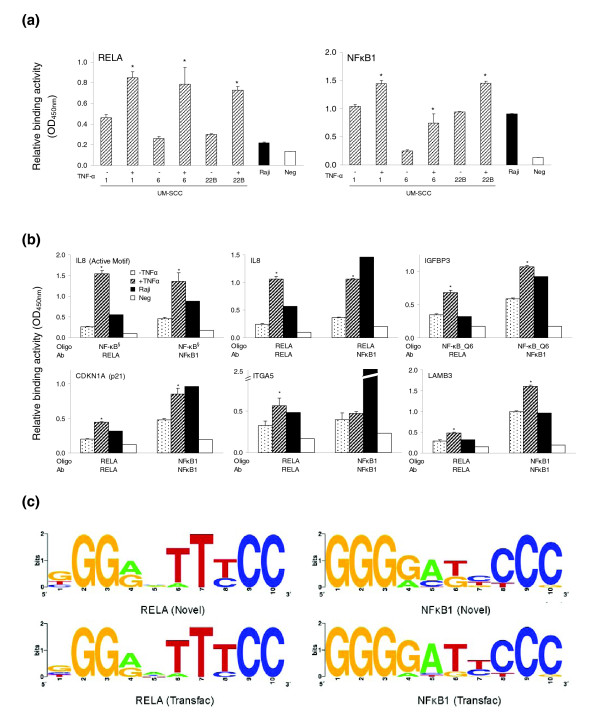
Binding activity and motif logo of RELA and NF*κ*B1. **(a) **The basal and inducible binding activity of RELA or NF*κ*B1 were tested using TransAM NF*κ*B family kit in UM-SCC 1, 6 and 22B cells after TNF-*α *(2000 units/ml) treatment. 'Raji' and 'Neg' represent positive and negative controls, respectively. **(b) **Binding activity of RELA and NF*κ*B1 in the promoter of NF-*κ*B target genes. The promoter sequences with putative RELA or NF*κ*B1 binding sites were synthesized as 50-mer oligos and biotin labeled, and the assays were performed using TransAM flexi NF*κ*B family kit. *P < 0.05 compared with the control (*t *test). **(c) **Motif logos of RELA and NF*κ*B1 were generated from 202 and 151 genes differentially expressed in UM-SCC with their putative binding sites, respectively (upper panels). Motif logos of RELA and NF*κ*B1 from Transfac were included for the comparison (lower panels).

The binding activities of individual subunits of NF-*κ*B, such as RELA and NF*κ*B1, to synthetic oligonucleotides equivalent to predicted sequences of promoters of selected genes were quantified using a commercially available binding assay, as described in Materials and methods. NF-*κ*B family TF assays were performed for three UM-SCC cell lines (Figure 7a). All cell lines exhibited constitutively active RELA or NF*κ*B1 binding activities, which were induced further by TNF-α (Figure 7a). To dissect the specific binding activity of each NF-*κ*B subunit to their cognate promoter sequences as predicted above, we performed NF-*κ*B binding assays using the promoter-specific DNA oligonucleotides. We observed similar constitutive and inducible binding activities for the *IL8 *promoter sequence by both RELA and NF*κ*B1 in the control oligonucleotide generated by Active Motif (containing only the 10 bp core sequence of the RELA binding motif, Figure 7b, upper left panel), or using oligonucleotides containing a larger 50 bp sequence that included the RELA binding motif (Figure 7b, upper middle panel). These data are consistent with the previous experimental results using electrophoretic mobility shift assay and chromatin immunoprecipitation (ChIP), showing that RELA/NF*κ*B1 heterodimers are involved in the binding of the *IL8 *promoter, leading to target gene expression [6,14]. Next, we tested the binding activity on the promoters of less studied NF-*κ*B targeted genes. The promoter of *IGFBP3 *was predicted to contain NF-*κ*B_Q6 binding motifs, which can not discriminate the binding activities of specific NF-*κ*B subunits, and our results support the prediction (Figure 7, upper right panel). In promoters of the remaining three genes, both RELA- and NF*κ*B1-specific binding motifs were predicted. In most cases, we observed the basal and TNF-α-induced binding activities of RELA or NF*κ*B1 (Figure 7, lower panels). Our experimental data confirmed the predicted binding motifs of selected genes tested.

Based on the predicted binding activity, we generated a logo of RELA or NF*κ*B1 binding motifs predicted by COGRIM from 202 and 151 genes, respectively (Figure 7a, upper panels). Our logos of RELA and NF*κ*B1 binding motifs are very similar to their consensus sequences and logos generated from position weighted matrices (PWMs) of Transfac 8.4: GGRRATTTCC (RELA) and GGGGATYCCC (NF*κ*B1), where underlined sequences represent core sites, and R = A or G, and Y = C or T.

## Discussion

In this study, we used a newly developed COGRIM statistical model to systematically define NF-*κ*B regulons of genes differentially expressed by UM-SCC cells (Figures 1 and 2). These NF-*κ*B regulons are connected to networks and signal pathways, for which there is evidence of significant involvement in tumorigenesis (Figures 3 and 4, and Table 1). Our experimental data confirmed and validated computational and bioinformatic predictions for NF-*κ*B regulation and binding activity on the promoter sequences of a selection of these genes (Figures 5, 6, 7), indicating that NF-*κ*B family members function as important master controls of gene expression, coordinating action within networks and pathways that contribute to the malignant phenotype of UM-SCC. Our study revealed the power of a systems biology analysis using COGRIM modeling and IPA to identify molecular signatures at the global level that are modulated by functionally active TFs, interacting networks and signaling pathways.

This study is the first utilization of COGRIM to analyze a family of TFs in a human cancer system [47,53]. Previously, there have been limited genome-wide computational analyses of NF-*κ*B binding activity and regulated genes related to malignant phenotypes and genotypes, due to the complexity of NF-*κ*B regulatory mechanisms, heterogeneous cancer subtypes, and inherent limitations or biases in computational and experimental conditions. An important feature of the COGRIM model is the ability to computationally analyze complex transcriptional regulatory mechanisms by simultaneously integrating multiple large scaled data sources, in a principled and robust fashion without requiring *a priori *knowledge of the relative accuracy of each data source. This model-based strategy greatly improved the efficiency and accuracy of the elucidation of the functional and physical relationships among the TFs, pathways and networks. Although the linear model of expression used as a basis for COGRIM is an approximation of transcriptional regulation, it has proven to be effective in other investigations [54-56]. One potential limitation of COGRIM is that the TF activity f_jt _must be approximated by a proxy measure such as the expression level of the gene that codes for that TF. The predicted functions of TFs are confirmed with experimental results even when extensive ChIP binding data were not available [47].

As described previously [47], the COGRIM method includes a probabilistic model for each data source that addresses the inherent uncertainty within each data type. COGRIM is more than a simple extension of previous linear models in that it provides a principled mechanism for integrating sequence features with expression data for the prediction of target genes and can be further extended in several interesting directions in the presence of additional data sources. It should also be noted that although we have focused on TFs, the model would work equally well with regulatory factors that are not proteins but whose levels can be measured and whose binding sites can be identified (for example, microRNAs). COGRIM represents an initial step toward solving the problem of integrating available biological information in a principled fashion. Our belief is that this goal will best be accomplished by fitting large and flexible probability models that combine data from various experimental and compiled sources in a structured or multi-level framework. We anticipate that the model will become even more valuable as the accuracy and coverage of expression and sequence feature data improve.

Using COGRIM in this study, 748 putative NF-*κ*B target genes were identified, which consisted of 59% of 1,265 differentially expressed genes from microarray analysis in UM-SCC cells (Figure 1 and Additional data file 1). This ratio is slightly higher than the frequency of all predicted NF-*κ*B binding motifs calculated in vertebrates (approximately 50%, including human, mouse and rat data from the Genomatix promoter database), but is slightly lower than the frequencies of NF-*κ*B binding motifs predicted in the up-regulated gene clusters enriched with known NF-*κ*B related genes published previously (approximately 65-70% in B-C gene clusters) [14]. The prediction is consistent with the hypothesis and experimental data that NF-*κ*B regulated genes are over-represented in tumor associated gene signatures, especially in the up-regulated gene clusters [14]. Interestingly, the overall ratio of approximately 60% of differentially expressed genes in human UM-SCC cells is remarkably consistent with the approximate percentage of genes in murine squamous cell carcinoma restored to expression levels seen in non-malignant cells of syngeneic origin by inhibition of NF-*κ*B using an inducible mutant I*κ*B*α *[13]. Inhibition of NF-*κ*B and target genes in this murine model was accompanied by decreased proliferation, migration, cell survival, angiogenesis and tumorigenesis [13]. The murine NF-*κ*B modulated gene signature was independently associated with a gene signature associated with decreased prognosis in a large series of human HNSCC[43]. Together, these experimental and *in silico *analyses of expression profiling data in murine and human squamous cell carcinoma are consistent with involvement of NF-*κ*B as a key regulatory factor in global alterations in gene expression in squamous cell carcinoma.

The efficiency and accuracy of COGRIM prediction are also supported by cross validation with other experimental data from published literature, as well as with our experimental results from UM-SCC cells upon TNF-*α *stimulation or siRNA knock down of NF-*κ*B (Figures 5, 6, 7) [14]. Among the 748 genes predicted as NF-*κ*B target genes, 75 of them (10%; in bold in Additional data file 1) overlapped with approximately 600 NF-*κ*B target genes published previously by the three websites described in the Materials and methods, indicating most of the predicted genes represent novel NF-*κ*B target genes. Additionally, only 16 genes of the list of 1,265 'known NF-*κ*B genes' based on these websites were excluded from our predicted gene list, due to low probability scores by COGRIM modeling (data not shown). Among the 16 genes, 3 were previously implicated in HNSCC and other cancers, namely *AREG *(amphiregulin), *MMP14*, and *MYC*. After searching the original references, we found the reference for *AREG *was incorrectly cited. For *MMP14*, a NF-*κ*B binding motif was observed in the promoter; however, it is located at -1,165 bp from the transcriptional stating site, which is outside the proximal promoter sequence defined in this study. The reference for *MYC *was published in 1990, for which the consensus sequence of the NF-*κ*B binding motif cited does not exist in the most updated human genome sequence (data not shown). However, there are several references suggesting other subunits of NF-*κ*B could be involved in the regulation of *MYC*, including evidence that the RELB/p52 complex can directly bind to the *MYC *gene promoter [24]. However, the PWM of RELB/p52 binding motifs has not been well established, and the computation in this study did not include RELB/p52. There have been reports about possible involvement of cREL in *MYC *gene expression but without discussing detailed mechanisms [24,57]. Thus, the COGRIM modeling in this study successfully predicted 82% (75/91) of known NF-*κ*B genes identified. The few cases of failed prediction could be either due to errors in literature citations, or because the location of the NF-*κ*B binding site is outside of the promoter sequence boundary selected for this study.

We experimentally validated a selected subset of predicted NF-*κ*B genes involved in signal pathways, using TNF-*α *and siRNA as tools. We showed that TNF-*α *significantly enhanced gene expression of 15 genes selected from the prediction, where 6 are novel NF-*κ*B targets, including *IL1R2*, *ALDH1A3*, *ITGA2*, *ITGA5*, *LAMA3 *and *LAMB3 *(Figure 5 and Additional data file 1). To date, we have experimentally tested expression of a total of 47 genes in response to TNF-*α *(Figure 5) [14], where 41 genes were identified as NF-*κ*B target genes by COGRIM, of which 23 are novel. Previously, there have been several experimental studies attempting to globally investigate NF-*κ*B binding activity and regulated gene expression, including RELA binding activity throughout human chromosome 22 [58], and TNF-*α*-induced NF-*κ*B target gene expression in HeLa cells [59,60], U937 monocytic cells [61], lipopolysaccharide-stimulated human peripheral blood mononuclear cells [62], and THP.1 cells transfected with IKK*γ *[63]. Under TNF-*α *stimulation, 767 genes (*P *< 0.05) or 343 genes (*P *< 0.01) were differentially expressed by HeLa cells [60], 348 genes exhibited NF-*κ*B binding activity in U937 cells [61], and 79 or 72 genes were identified as NF-*κ*B regulated and responsive to lipopolysaccharide in macrophages [62,63]. Among these gene lists, a total of 88 genes were confirmed as NF-*κ*B-regulated genes and overlapped with our gene list (Additional data file 1), where 25 genes were identified as known NF-*κ*B genes listed by the three websites previously mentioned. These experimental data also cross-validate 63 putative NF-*κ*B target genes identified by our analysis.

We noticed different kinetics in the expression of gene subsets induced by TNF-*α *in UM-SCC cell lines. The responsive kinetics of many of the novel NF-*κ*B target genes are either slowly induced, or induced and sustained without rapid decrease (Figure 5), in contrast to the typical TNF-*α*-induced early response gene, as observed for cytokines (*IL1A*, *IL1B*, *IL1RN*, *CSF2 *and *VEGFC*) and a NF-*κ*B family member (*REL*). Different kinetics of gene expression in response to TNF-*α *treatment has been noticed previously [59], where rapid oscillatory responses could be due to TNF-*α*-mediated phosphorylation, degradation and re-synthesis of I*κ*B*α*, in contrast to that of I*κ*B*β *and I*κ*B*ε*, which mediate prolonged stimulation [64]. It has also been reported that the TNF-*α*-induced early response pattern is seen often in genes with conserved promoter regions [59]. This observation supports the hypothesis that the genes with typical early response inducible patterns are those with evolutionarily conserved functions involved in the first line of defense, where a quick reaction and termination mechanism is needed. The genes with the slower induction patterns are involved in functions such as adhesion and cell structure, where slower and persistent responses are necessary.

We also observed that the predicted NF-*κ*B regulons are not uniformly distributed in the subgroups of UM-SCC cells (Figure 2), and more genes with predicted RELA-specific regulatory motifs were observed in the up-regulated gene groups with wild-type p53-deficient status (Figure 2c). This observation is in good agreement with the general consensus and experimental data regarding the positive regulatory role of RELA in controlling oncogenic gene expression [2,25,65], and is consistent with our observation that in UM-SCC cells with wild-type p53-deficient status a cluster of NF-*κ*B regulated genes is over-expressed [14,35]. The experiments knocking down *RELA *or *NFκB1 *elucidated NF-*κ*B-mediated specific regulatory mechanisms (Figure 6), and provided data consistent with the previous findings that the basal expression of most of the NF-*κ*B-regulated genes depends on both RELA and NF*κ*B1 (p65/p50 heterodimer). Negative regulation of NF*κ*B1 compared to the basal gene expression was consistent with a repressive function associated with p50 homodimers [1,2]. Furthermore, the binding activities of RELA and NF*κ*B1 were confirmed in the promoter regions of a typical NF-*κ*B target gene, namely *IL8 *(which served as the positive control), in the promoters of atypical NF-*κ*B target genes, namely *IGFBP3 *and *CDKN1A*, and in the promoters of novel NF-*κ*B target genes, namely *ITGA5 *and *LAMB3 *(Figure 6b). Interestingly, *CDKN1A *is also a p53 target gene with important function in the control of the cell cycle and apoptosis, and *IGFBP3 *is a p63 target gene involved in the IGF signaling pathway [66]. Our data provide computational and experimental evidence consistent with potential cross-talk between the two important pathways, namely NF-*κ*B and p53/p63, through which target genes could be transcriptionally regulated by either or both TFs.

The NF-*κ*B target genes identified were connected by networks and functioned as regulons under direct interaction or close regulation by RELA (Figure 3a,b), or NF*κ*B1 (Figure 3c,d). These gene groups included many known NF-*κ*B target genes with confirmed NF-*κ*B binding sites, such as *CCDN1*, *CSF1*, *CSF2*, *ELF3*, *ICAM1*, *IL1A*, *IL1B*, *IL1RN*, *IL2RA*, *IL6*, *IL8*, and *VIM*. Interestingly, most of these known NF-*κ*B target genes appear in the networks with RELA (Figure 3a,b), and are less significantly associated with NF*κ*B1 (Figure 3c,d). This observation is consistent with the fact that RELA contains the functional transactivation domain for mediating gene transcription [2,25,65]. In addition, even fewer NF-*κ*B target genes were connected with NF*κ*B1 in cells with wild-type p53-deficient status (Figure 3c); this subgroup of cells over-expressed genes with a high prevalence of RELA regulation (Figure 2c). In this subgroup of cells (Figure 3c), more genes were connected to peroxisome proliferator-activated receptor gamma (PPARG), a member of the nuclear hormone receptor subfamily. PPARG is able to form heterodimers with retinoid X receptors, affect RELA cytoplasmic distribution and negatively regulate inflammatory responses [67]. Interestingly, in this network, PPARG is also linked with PPAR binding protein, a PPAR co-activator with the ability to bind to DNA and p53 protein [68,69]. Our and other data provide computational and experimental evidence consistent with potential cross-talk between the two important pathways, namely NF-*κ*B and p53/p63, through which target genes could be transcriptionally regulated by either or both TFs.

The up-regulated NF-*κ*B target genes are enriched in important signal pathways implicated in most cancers, including leukocyte extravasation, inositol phosphate metabolism, and xenobiotic metabolism pathways (Table 1 and Figure 4a). The pathways identified are consistent with previous evidence from studies by us and others that NF-*κ*B promotes proinflammation, pro-angiogenesis, cell adhesion and migration through up-regulation of genes involved in these pathways [4,6-16,70,71]. The inositol phosphate metabolism pathway consists of molecular components of PI3K and protein kinase C pathways, both important signal pathways implicated in promoting tumorigenesis, especially in epidermis and epithelia [72-75]. The involvement of NF-*κ*B with the down-regulated genes has been less studied; in this study, genes in Wnt/*β*-catenin and TGF-*β *pathways were down-regulated in all tumor cells through regulation in association with all NF-*κ*B family members (Figure 4b). The Wnt/*β*-catenin signaling pathway includes many negative regulators of cell growth and survival, and the down-regulation of these genes has been shown to be the critical step in tumorigenesis in epidermis and epithelia [76]. Interestingly, the involvement of RELA and NF*κ*B1 in the Wnt/*β*-catenin pathway was not significant, suggesting other NF-*κ*B family members or NF-*κ*B-independent intermediates could be involved. The TGF-*β *signaling pathway is another negative regulatory pathway and the resulting deficiency has been demonstrated in HNSCC. Lu *et al*. [77] identified a defect of TGF-*β *receptor 2 (*TGFβR2*) and the related pathway that significantly contributes to HNSCC carcinogenesis and metastasis. Other signal pathways identified are more specific to the phenotypic and genotypic differences in UM-SCC cells resulting from different p53 statuses (Figure 4). The pathways related to IGF, integrins, receptor and intermediate signals (Ephrin receptor, NF-*κ*B, p38 MAPK, PPAR and PTEN, and cytokines (VEGF and GM-CSF are dominant in cells with mutant p53 status, which is consistent with either the loss of the negative regulation (PTEN and PPAR), or the suppression of NF-*κ*B and other signal pathways and genes by gaining or retaining p53 functions in cells with mutant p53 status [14,35]. For cells with wild-type p53-deficient status, down-regulated genes were only involved in the cell cycle:G2/M DNA damage checkpoint pathway, where RELA showed dominant effects (Figure 4b).

This study provides a strong link between NF-*κ*B regulons and related pathways identified by the systems biology approaches, consistent with many conclusions previously drawn from individual and classic biological experiments. The data from both computational and experimental strategies support the hypothesis that the malignant progression of HNSCC is due to, or leads to, multiple genetic and phenotypic defects, such as p53 mutation or underexpression [38,78], and aberrant activation of several major growth factor and cytokine receptor pathways, including the TNF receptor [16], IL1R [9,39], IL6R [31], EGFR [10], hepatocyte growth factor receptor/cMet [41], TGF-*β *receptor [77], and platelet-derived growth factor receptor [79] pathways. These receptors modulate multiple signal pathways, including aberrant activation of NF-*κ*B [6,7,19], AP1 [6,9], JAK/STAT [31], early growth response-1(EGR1) [37], casein kinase 2 [11], MAPK [15], PI3K [10,41], and BCL-XL/IAP associated apoptosis pathways [8]. Our previous report showed that the five major TFs - NF-*κ*B, STAT3, AP1, EGR1, and p53 - are specifically implicated in the unique gene signatures of UM-SCC cells [14], adding supporting evidence to current work that multiple transcriptional mechanisms and signal pathways control specific gene and pathway signatures that determine the malignant phenotypes and the heterogeneous characteristics in UM-SCC cells.

In interpreting our current study, we recognize that there are differences between cell lines and human tissues. However, many of our previous studies using these cell lines have led to the demonstration and confirmation of important molecular findings made with them in tumor tissue and serum specimens. These include the demonstration of alterations and the biological and clinical significance of NF-*κ*B activation and of multiple NF-*κ*B-regulated genes and cytokines expressed in HNSCC tumor specimens and serum [18,32,33,42,80,81], and the demonstration of an inverse relationship between NF-*κ*B and p53 nuclear localization and associated protein expression in tumors [35]. As a result, and to further examine the validity of the results of the bioinformatic analysis of the present study, we have recently undertaken a meta-analysis of 34 microarray datasets of HNSCC (approximately 80% from tissue specimens). Preliminary analyses are consistent with key observations from this study using UM-SCC cell lines, including that the molecules in, and/or regulated by, the NF-*κ*B and p53 signaling pathways are significantly enriched and related to HNSCC malignancy (B Yan *et al*., manuscript in preparation). Since there are many important differences between the tumor cell lines in culture and human tumor specimens, where the paracrine effects from fibroblasts and other host cells are missing, it will be important in future studies to integrate stromal cell gene and protein expression data with functional studies of potential networks involving these interactions.

## Conclusion

We successfully predicted NF-*κ*B regulons through COGRIM modeling and connected them into organized NF-*κ*B regulatory modules. This analysis revealed the concerted activation of NF-*κ*B target genes or gene products, many of them previously identified as unrelated molecules. The analysis of NF-*κ*B regulons established a complex interaction comprising novel or previously identified pathways and networks, where the molecular signatures were particularly associated with cells differing in p53 status. Our study identified pathway signatures related to UM-SCC cells in general for over-activated proinflammation, self-defense and inositol phosphate metabolism, as well as down-regulated Wnt/*β*-catenin, TGF-*β *and neuregulin pathways. RELA-controlled over-activation of *IL6 *signaling and down-regulated cell cycle:G2M checkpoint was specific for tumor cells with wild-type p53-deficient status. Up-regulated IGF signaling and multiple down-regulated pathways comprise the molecular signature for cells with mutant p53 status. Such molecular signatures composed of multiple pathways established the foundation for further global identification of biomarkers and therapeutic targets in HNSCC and other cancers with phenotypic and genetic heterogeneity related to p53 status or other abnormalities.

## Materials and methods

### Cell lines

Ten established HNSCC cell lines, UM-SCC 1, 5, 6, 9, 11A, 11B, 22A, 22B, 38 and 46, were obtained from the University of Michigan series of HNSCC (UM-SCC, Ann Arbor, MI, USA), as described previously [40]. The ten UM-SCC cell lines were obtained from eight HNSCC patients, representing aggressive malignancies derived from different anatomic sites. Many molecular alterations of these cell lines were confirmed in HNSCC tumors, including over-expression and activation of EGFR, IL1 and IL6 signal transduction pathways, mutation and altered activation of TFs p53, NF-*κ*B, AP-1, STAT3 and EGR1, over-expression of proinflammatory and proangiogenic cytokines and genes, and resistance to radiation and chemotherapies [6-9,14,15,18,37,43,44,49]. Human normal keratinocytes were obtained from four individuals (Cascade Biologics Inc., Portland, OR, USA), and cultured following the manufacturer's protocol. p53 mutation and expression status of UM-SCC cell lines were carried out with bidirectional genomic sequencing of exons 4-9, and confirmed with western blotting and immunohistochemistry [14]. Mutation of p53 was detected in six cell lines, UM-SCC 5, 11B, 22A, 22B, 38 and 46. Four cell lines, UM-SCC 1, 6, 9 and 11A, retained a wild-type p53 genotype [14].

### Microarray experiments and data analysis

The cDNA microarray chips containing 24K human elements were from NHGRI/NIH (Bethesda, MD, USA). The experimental procedures and data are available at Gene Expression Omnibus [82] (Series accession number GSE10774 Microarray experimental design, data collection and analyses were as described previously [83]. The subgroups of UM-SCC cells were identified according to microarray data analysis using principle components analysis and hierarchical clustering analysis. Subgroup 1 included five UM-SCC lines with mutant p53 status, and subgroup 2 was defined as wild-type p53-like status and included four UM-SCC lines with wild-type p53 sequence but deficient expression, plus 11B cells, which express a transcriptionally deficient mutant p53 and the same gene signature [14,38]. Thus, in this study, the definition 'wild-type p53-like status' is changed to 'wild-type p53-deficient' based on data showing that p53 expression and function is deficient in this group of cells in the absence of mutations found in promoter and coding sequences [38]. We identified differentially expressed genes among human normal keratinocytes and UM-SCC subgroups that satisfied the following criteria: two-fold and above change of average gene expression in the ten UM-SCC cell lines or in either subgroup with different p53 statuses when compared with the average gene expression of human normal keratinocytes. These criteria resulted in 1,265 genes for the following analyses (Figure 1).

### Extraction of promoter sequences and TF matrices

The promoter sequences of the 1,265 differentially expressed genes were extracted using DBTSS [84] and Genomatix suite 3.4.1 [85]. The proximal promoter of each gene was set to 1,000 bp upstream and 300 bp downstream of the transcriptional start site. PWMs for NF-*κ*B binding sites were derived from Transfac 8.4 [86]. PWMs for five NF-*κ*B binding sites were used, including: RELA/p65 (NFKAPPAPP65), NF*κ*B1/p50 (NFKAPPABP50), cREL, NF*κ*B_Q6 and NF*κ*B_Q6_01.

### COGRIM modeling

We applied COGRIM [47] to identify NF-*κ*B gene regulons by integrating the data sources from differentially expressed gene profiles, the related promoter sequences, NF-*κ*B binding activities in UM-SCC cell lines, and PWMs for NF-*κ*B binding sites (Figure 1). Briefly, COGRIM is a Bayesian hierarchical model with a Markov chain Monte Carlo implementation that is able to integrate heterogeneous biological data and avoid an *ad hoc*, stage-wise analysis by simultaneously modeling gene clustering and the strength of the contribution from each of the data sources [47].

The central goal of the COGRIM procedure is to infer the correct set of gene-TF associations, which are mathematically formulated as indicator variables C_ij_. The variable C_ij _= 1 if gene *i *is regulated by TF *j*, and C_ij _= 0 if there is no regulation of gene *i *by TF *j*. The first level of COGRIM incorporated our gene expression data into the inference for each C_ij _by specifying the observed gene expression g_it _as a linear function of TF activity, f_jt_.

$$\begin{array}{cc} g_{it}=\alpha_{i}+\sum_{j=1}^{J}\beta_{j}C_{ij}f_{jt}+\varepsilon_{it} & \varepsilon_{it}\sim Normal(0,\delta^{2}) \end{array}$$

Note that only TFs with connections to gene *i *(that is, C_ij _= 1) are allowed to influence the expression of gene *i *in the equation above. This also means that *α*_i _can be interpreted as the baseline expression for gene *i *in the absence of regulation by known TFs (that is, C_ij _= 0 for all TFs *j*), whereas *β*_j _can be considered as the linear effect of TF *j *on target gene expression. As mentioned above, we have one additional class of data for inferring a particular gene-TF association C_ij_: promoter element data m_ij_. The probability of a binding site for TF *j *in the upstream region of gene *i*, m_ij_, is calculated using TESS [87]. We use m_ij _as the prior probability for the variable C_ij _as described previously [47]. Note that we have a choice between two proxies for the TF activity f_jt_: the expression of the gene that encodes TF *j *or the experiment results from NF-*κ*B DNA binding assays (as described in the NF-*κ*B DNA binding assay section). In our analysis, we compare the results based on these two different proxies for TF activity.

COGRIM identifies putative NF-*κ*B gene targets (regulons) that represent a set of genes (748; Figure 1) regulated by the five NF-*κ*B PWMs described previously by Transfac 8.4. The probability cutoff scores (0.8) were proposed to infer putative NF-*κ*B gene targets based on the comparisons to background models. These genes included 11 known NF-*κ*B target genes with slightly lower scores than the cutoff. The known NF-*κ*B target genes were defined by PubMed publications and available NF-*κ*B websites [88-90].

### Gene Ontology annotation

Gene Ontology annotation was performed using Onto-Express software [91]. A hypergeometric distribution was used to calculate the significance values in the probability model. Corrected *P *< 0.05 was used as the cutoff value in this study.

### Analysis of networks and pathways

The putative NF-*κ*B regulated genes identified from COGRIM were imported into IPA 5.0 (Ingenuity Systems Inc, Mountain View, CA, USA) according to the Ingenuity Pathways Knowledge Base (IPKB), where each interaction in IPKB is supported by the underlying publications and structured functional annotation [92]. Statistical scores were then assigned to rank the resulting networks and pathways using Fisher's right tailed exact test, where the significant networks (*P *< 0.001) and pathways (*P *< 0.05) were selected. IPA allows comparison of gene networks and pathways generated by different sets of input genes, or under the regulation of different TFs. In this study, we input predicted RELA or NF*κ*B1 target gene sets from Figure 2b into IPA, and used *RELA *or *NFκB1 *as override genes to identify RELA or NF*κ*B1 directly interacting genes and networks.

### TNF-*α*-induced gene expression in UM-SCC cells

TNF-*α*-induced gene expression was studied in cultured UM-SCC 6 cells treated with or without TNF (2,000 units/ml; Knoll Pharmaceutical Company, Whippany, NJ, USA). Total RNAs were isolated using a Trizol reagent (Invitrogen, Carlsbad, CA, USA) and RNeasy Mini Kit (QIAGEN, Valencia, CA, USA) combined method at indicated time points. cDNA synthesis was performed by using a High-Capacity cDNA Archive Kit (Applied Biosystems, Foster City, CA, USA). PCR was performed together with endogenous eukaryotic 18S ribosomal RNA (18S) as the control using Assays-on-Demand™ Gene Expression Assay (Applied Biosystems), as previously described [14,35]. Relative quantification of the expression was calculated by normalizing the target gene signals with the 18S endogenous control. An arbitrary unit was calculated after setting C_T _to 40 as undetectable expression and used for normalization.

### Knocking down NF-*κ*B RELA and NF*κ*B1 by siRNA

The knockdown of *RELA *and *NFκB1 *mRNA was performed by using siRNA (ON-TARGETplus SMARTpool; Dharmacon, Lafayette, CO, USA). UM-SCC 6 cells were seeded in 6-well plates at 1 × 10^5^/well. At 50-60% confluency (24 h later), cells were transfected with 50 nM of a mixture of four siRNA oligos directed against human *RELA *or *NFκB1 *designed by Dharmacon, or 50 nM of a non-silencing control siRNA (QIAGEN), using 1:200 Lipofectamine 2000 (Invitrogen) in Opti-MEM I Reduced Serum Medium (Invitrogen) for 5 h. At 24 h and 48 h post-transfection, cells were harvested in Trizol for RNA isolation (Invitrogen).

### NF-*κ*B DNA binding assays

Cultured UM-SCC cells were treated with TNF-*α *for 30 minutes. Nuclear fractions were harvested from cells using the nuclear extraction kit following the manufacturer's suggestions (Active Motif, Carlsbad, CA, USA). The protein concentration was determined using the BCA method (Pierce, Rockford, IL, USA). NF-*κ*B DNA binding activity was quantitatively assessed using the TransAM NF-*κ*B family TF assay kit (Active Motif) per the manufacturer's protocol. To each well containing a NF-*κ*B consensus binding site (5'-GGGACTTTCC-3'), 10 *μ*g of nuclear extract in cell binding and cell lysis buffer were added in each well in triplicates. We used 5 *μ*g of nuclear extract of Raji cells (a Burkitt's lymphoma cell line) as the positive control (Active Motif). To assess DNA binding specificity, excess wild-type NF-*κ*B consensus oligonucleotide was added (20 pmol/well) to compete for the binding, as compared with other wells to which was added an inactive mutated consensus oligonucleotide. Plates were washed using the ELx50 strip washer (Bio-Tek, Winooski, VT, USA), and the absorbance was measured at 450 nm by *μ*Quant ELISA microplate reader (Bio-Tek).

To dissect the specific binding activity of each NF-*κ*B subunit for their cognate promoter sequences as predicted above, we performed NF-*κ*B binding assays using promoter-specific DNA oligonucleotides in TransAM flexi NF-*κ*B family kit (Active Motif). The 50 bp oligonucleotides contains a 10 bp core consensus sequence of a RELA binding motif of the *IL8 *promoter generated by Active Motif as the positive control (5'-biotin-GGGCCATTTACCGTAAGTTATGTAACGCGCCTGGGAAATTCCACTCAACT-3'; the underlined sequence is the NF-*κ*B binding consensus). We also generated 50 bp oligonucleotides based on the predicted RELA binding motif flanked with the natural sequences of promoters of: *IL8*, 5'-biotin-CCCTGAGGGGATGGGCCATCAGTTGCAAATCGTGGAATTTCCTCTGACAT-3' for the RELA binding site; *CDKN1A*, 5'-biotin-ACTGAGCCTTCCTCACATCCTCCTTCTTCAGGCTTGGGCTTTCCACCTTT-3' for the RELA binding site, 5'-biotin-AGGTGAATTCCTCTGAAAGCTGACTGCCCCTATTTGGGACTCCCCAGTCT-3 for the NF*κ*B1 binding site; *ITGA5*, 3'-biotin-CTCCGCCCACCAGAGGTGATTCCTTTCCTCATTAGGAAATTCTCCGCTCC-5' for the RELA binding site, 3'-biotin-AGAACCCAGGCACCCGGCGGCCCCGGAAGGCAAGGGGGAATCCCAGTTGG-5' for the NF*κ*B1 binding site; *LAMB3*, 3'-biotin-ACTTGTGGTCAGGTCTGTTTTCTGGCCCTCCAGG CGGGCATTCCTGCCTA-5' for the RELA binding site, 3'-biotin-GGTGAGGCTGTTGTTTAAAAACCTGGAGCCGGGAGGGGAGACCCCCACAT-5' for the NF*κ*B1; *IGFBP3*, 3'-biotin-GGCAAGCGTCCAATTTCAACAGCGTTCAGGAAAGTCTCCTCCCGCGGAGG-5' for the NFkB_Q6 (a NF-*κ*B PWM of Transfac 8.4) binding motif. The oligonucleotides were about 50 bp in length, each with gene specific promoter sequences, and were custom synthesized and biotinylated at the far end from the NF-*κ*B consensus sites by the Midland Certified Reagent Company (Midland, TX, USA). Ten micrograms of nuclear extract, 50 *μ*l binding buffer, and 1 pmol of biotinylated oligonucleotide were incubated for 30 minutes at room temperature prior to placement in a well on strepavidin-coated 96-microtiter plates. The binding experiments were performed following the protocol provided by TransAM NF-*κ*B family TF assay kit as described.

## Abbreviations

AKT, v-akt murine thymoma viral oncogene homolog 1; AP1, activating protein-1; ChIP, chromatin immunoprecipitation; COGRIM, Clustering Of Gene Regulons using Integrated Modeling; EGFR, epidermal growth factor receptor; EGR1, early growth response-1; ETS, v-ets erythroblastosis virus E26 oncogene homolog (avian); GM-CSF, granulocyte-macrophage colony-stimulating-factor; HNSCC, head and neck squamous cell carcinoma; IGF, insulin-like growth factor; I*κ*B, inhibitor kappaB; IKK, inhibitor-kappaB kinase; IL, interleukin; IL-R, interleukin receptor; IPA, Ingenuity Pathway Analysis; IPKB, Ingenuity Pathways Knowledge Base; MAPK, mitogen-activated protein kinase; NF-*κ*B, nuclear factor kappaB; PI3K, phosphatidylinositol 3-kinase; PPAR, peroxisome proliferator-activated receptor; PTEN, phosphatase and tensin homolog; PWM, position weighted matrix; siRNA, small interfering RNA; STAT, signal transducer and transcription factor; STAT3, signal transducer and transcription factor 3; TF, transcription factor; TGF, transforming growth factor; TNF, tumor necrosis factor; UM-SCC, University of Michigan Cell Lines Series of Head and Neck Squamous Cell Carcinoma; VEGF, vascular endothelial growth factor.

## Authors' contributions

BY, GC and ZC conceived and designed the bioninformatic and analytic strategies and experiments. GC, STJ and CJS originally developed the COGRIM model. BY prepared micorarray and related promoter data for caculation through COGRIM modeling. GC performed mathematical caculation through COGRIM modeling. BY performed data analysis after mathematical caculation using IPA and other bioinformatic tools. BY, KS, XY and ZC contributed to the experimental designs. BY, KS and XY wrote experimental protocols, and performed experiments and data analyses. BY prepared the final figures and tables. ZC, BY, CVW, GC, STJ and CJS contributed to the writing and discussion of the paper.

## Additional data files

The following additional data are available with the online version for this paper. Additional data file 1 lists NF-*κ*B target genes differentially expressed in UM-SCC cell lines. Additional data file 2 lists Gene Ontology annotations for NF-*κ*B target genes in wild-type p53-deficient, mutant p53 and wild-type plus mutant p53 subsets of UM-SCC cells. Additional data file 3 shows network lists generated by IPA based on gene sets regulated by NF-*κ*B subunits RELA/p65 and NF*κ*B1/p50.

## Supplementary Material

Additional data file 1 NF-*κ*B target genes differentially expressed in UM-SCC cell lines. Click here for file

Additional data file 2 Gene Ontology annotations for NF-*κ*B target genes in wild-type p53-deficient, mutant p53 and wild-type plus mutant p53 subsets of UM-SCC cells. Click here for file

Additional data file 3 Network lists generated by IPA based on gene sets regulated by NF-*κ*B subunits RELA/p65 and NF*κ*B1/p50. Click here for file
