# Supplementary material for: Systems biology-defined NF-κB regulons, interacting signal pathways and networks are implicated in the malignant phenotype of head and neck cancer cell lines differing in p53 status
Source: Genome Biol. 2008 Mar 11;9(3):R53. doi: 10.1186/gb-2008-9-3-r53 (PMC2397505; doi:10.1186/gb-2008-9-3-r53)
Supplement: Additional data file 1 — NF-κB target genes differentially expressed in UM-SCC cell lines. [file gb-2008-9-3-r53-S1.pdf]

Supplemental Table S1. Differentially expressed genes with predicted NF-κB regulation in HNSCC

Symbol	Gene description	Fold change [a]			NF-κB binding matrices (pwm) [b]				
		ΔT	ΔW	ΔM	RELA	NFκB1	cRel	Q6_1	Q6
AARS	alanyl-tRNA synthetase	2.28	2.13	2.44		h		h	h
**ABCA1**	**ATP-binding cassette, sub-family A (ABC1), member 1**	0.27	0.24	0.31	h	h		h	h
ABCC5	ATP-binding cassette, sub-family C (CFTR/MRP), member 5	2.28	0.88	3.68		h			
ABCG2	ATP-binding cassette, sub-family G (WHITE), member 2	1.92	2.86	0.98	h	h			
ABLIM1	actin binding LIM protein	1.83	1.13	2.52		h			
ACAA2	acetyl-Coenzyme A acyltransferase 2	1.7	2.29	1.12		h			
ACAS2	acetyl-Coenzyme A synthetase 2 (ADP forming)	0.37	0.4	0.35					h
ACAT2	acetyl-Coenzyme A acetyltransferase 2 (acetoacetyl Coenzyme A thiolase)	0.46	0.48	0.45	h			h	
ACLY	ATP citrate lyase	0.3	0.34	0.27	h		h		h
ACSL1	fatty-acid-Coenzyme A ligase, long-chain 2	0.6	0.42	0.78				h	
ACSL3	fatty-acid-Coenzyme A ligase, long-chain 3	0.58	0.68	0.49			h		
ACSL4	fatty-acid-Coenzyme A ligase, long-chain 4	0.42	0.48	0.37	h		h	h	h
ACSL5	fatty-acid-Coenzyme A ligase, long-chain 5	2.18	3.27	1.1	h	h	h	h	h
ACTG2	actin, gamma 2, smooth muscle, enteric	0.55	0.69	0.41				h	
ACTN3	actinin, alpha 3	0.49	0.54	0.44	h	h	h	h	
ADAM17	a disintegrin and metalloproteinase domain 17	0.59	0.44	0.74				h	h
**ADAM19**	**a disintegrin and metalloproteinase domain 19 (meltrin beta)**	0.35	0.42	0.27			h		
ADAM8	a disintegrin and metalloproteinase domain 8	0.35	0.24	0.47		h		h	
ADFP	adipose differentiation-related protein	0.37	0.43	0.31	h	h	h	h	h
ADPRTL3	ADP-ribosyltransferase (NAD+; poly (ADP-ribose) polymerase)-like 3	4.52	5.56	3.48	h			h	
**AGER**	**advanced glycosylation end product-specific receptor**	0.5	0.48	0.51		h			
AGPAT2	1-AGP acyltransferase 2	1.71	1.17	2.24		h			h
AGR2	anterior gradient 2 homolog (Xenepus laevis)	10.17	6.3	14.04	h		h	h	h
**AGXT**	**alanine-glyoxylate aminotransferase**	0.57	0.65	0.48	h		h		
AKAP12	A kinase (PRKA) anchor protein (gravin) 12	3.5	5.92	1.08	h	h	hm		
AKNA	AT-hook transcription factor AKNA	2.29	2.5	2.08	h		h		
AKR1C3	aldo-keto reductase family 1, member C3	3.98	0.88	7.09				h	
**AKT1**	**v-akt murine thymoma viral oncogene homolog 1**	0.52	0.68	0.37		hm	h		h
ALB	albumin	1.86	2.85	0.86	h	h		h	h
ALDH1A2	aldehyde dehydrogenase 1 family, member A2	2.12	1.18	3.06	h		h	h	h
ALDH1A3	aldehyde dehydrogenase 1 family, member A3	2.65	2.9	2.39	h	h			
ALDH3B2	aldehyde dehydrogenase 3 family, member B2	2	1.89	2.1	h	h		h	
ALDH4A1	aldehyde dehydrogenase 4 family, member A1	0.58	0.45	0.7		hm			
ALDH5A1	aldehyde dehydrogenase 5 family, member A1	2.03	2.18	1.88		h			
ALEX1	ALEX1 protein	0.4	0.42	0.39		h			

ALEX2	armadillo repeat protein ALEX2	0.16	0.18	0.15	h		h	h	h
ALP	alpha-actinin-2-associated LIM protein	2.36	2.12	2.6	h	h	h	h	h
AMY2A	amylase, alpha 2A; pancreatic	0.45	0.42	0.49	h		h	h	h
**ANGPT1**	**angiopoietin 1**	0.37	0.4	0.35					hm
ANK1	ankyrin 1, erythrocytic	10.39	0.91	19.86		h		h	
ANKT	nucleolar protein ANKT	2.53	2.26	2.8	hm				
ANPEP	alanyl (membrane) aminopeptidase	0.11	0.1	0.11		h			
AOX1	aldehyde oxidase 1	0.18	0.18	0.18		h			
AP3B1	adaptor-related protein complex 3, beta 1 subunit	0.65	0.8	0.27	h		h	h	
APBA2BP	amyloid beta (A4) precursor protein-binding, family A, member 2 binding protein	1.96	2.01	1.91	h		h	h	h
APBB2	amyloid beta (A4) precursor protein-binding, family B, member 2 (Fe65-like)	0.6	0.48	0.72		hm	hm	hm	hm
APIP	APAF1 interacting protein	2.01	2.17	1.84	hm			hm	
APRIN	Androgen-induced proliferation inhibitor	1.85	1.65	2.06		hm		hm	hm
**CENTD3**	centaurin, delta 3	2	2.44	1.56		h			
ARGBP2	Arg/Abl-interacting protein ArgBP2	2.01	1.12	2.9			h	h	h
ARHGEF2	rho/rac guanine nucleotide exchange factor (GEF) 2	0.49	0.53	0.45			h		
ARID3Aß	AT rich interactive domain 3A (BRIGHT- like)	1.65	2.21	1.09	h		h	h	
ARL6IP	ADP-ribosylation factor-like 6 interacting protein	2.24	2.47	2.01			hm	hm	
ARL7	ADP-ribosylation factor-like 7	0.33	0.39	0.27	hm	hm	hm		
ARMET	arginine-rich, mutated in early stage tumors	0.34	0.42	0.26	h	h		h	
ARTS-1	type 1 tumor necrosis factor receptor shedding aminopeptidase regulator	2.04	0.9	3.17	h	h		h	
**ASS**	**argininosuccinate synthetase**	0.43	0.31	0.54	h	h		h	
**ATF3**	**activating transcription factor 3**	2.14	1.86	2.41		h			
ATP1B3	ATPase, Na^+/K^+ transporting, beta 3 polypeptide	2.27	0.79	3.75		h	hm	h	
ATP2A2	ATPase, Ca^{++} transporting, cardiac muscle, slow twitch 2	0.51	0.55	0.47	hm	hm		hm	
AXIN1	axin	2.16	1.55	2.77		h			
B3GNT6	UDP-GlcNAc:betaGal beta-1,3-N-acetylglucosaminyltransferase 6	0.54	0.65	0.43	h	h		h	h
B4GALT1	UDP-Gal:betaGlcNAc beta 1,4- galactosyltransferase, polypeptide 1	0.45	0.53	0.37		hm			
B4GALT5	UDP-Gal:betaGlcNAc beta 1,4- galactosyltransferase, polypeptide 5	2.03	1.95	2.11	h				h
BAK1	BCL2-antagonist/killer 1	0.55	0.49	0.61					h
BAP29	B-cell receptor-associated protein BAP29	0.44	0.41	0.46		h		h	
BATF	basic leucine zipper transcription factor, ATF-like	1.59	1.08	2.09		h	hm	hm	
BCAR3	breast cancer anti-estrogen resistance 3	0.51	0.69	0.33		h			
BCAT1	branched chain aminotransferase 1, cytosolic	1.91	2.77	1.06		h			
**BCL10**	**B-cell CLL/lymphoma 10**	0.56	0.63	0.49		h		h	
BDG29	BDG-29 proten	1.74	1.37	2.11			hm		
BENE	BENE protein	0.57	0.42	0.72		h			
BEX2	Brain expressed X-linked 2	1.68	1.26	2.11					h
BF	B-factor, properdin	2.53	1.82	3.23	hm	hm	hm	hm	hm

**BIRC2**	**baculoviral IAP repeat-containing 2**	3.07	5.31	0.83	h				
**BMI1**	**B lymphoma Mo-MLV insertion region (mouse)**	0.5	0.53	0.47		hm			
BMP2K	BMP2 inducible kinase	0.57	0.68	0.47	h		h	h	h
**BMP4**	**bone morphogenetic protein 4**	0.48	0.36	0.6	h	h	h		h
BNC	basonuclin	0.39	0.36	0.42		h			
BOK	Bcl-2-related ovarian killer protein-like	0.39	0.42	0.36	h	h		h	
BPGM	2,3-bisphosphoglycerate mutase	0.78	0.42	1.13				h	h
BRF2	BRF2, subunit of RNA polymerase III transcription initiation factor, BRF1-like	1.53	0.97	2.1				h	
BRMS1	breast cancer metastasis-suppressor 1	1.84	1.64	2.03	hm	hm	h	h	h
BST2	bone marrow stromal cell antigen 2	2.32	1.11	3.53	h	h	h	h	h
BTG2	BTG family, member 2	0.72	0.44	1				h	
BTG3	BTG family, member 3	0.57	0.49	0.66	h			h	
C11orf23	chromosome 11 open reading frame 23	1.92	1.99	1.85	hm		hm	hm	hm
C11orf9	chromosome 11 open reading frame 9	1.53	1.07	1.99	h	h	h	h	
C1QTNF6	C1q and tumor necrosis factor related protein 6	1.57	2.06	1.07	h	h	h	h	h
C1S	complement component 1, s subcomponent	3.21	2.8	3.62			h		
C20orf10	clg01 protein	0.23	0.26	0.2	h	h	h		
C20orf11	chromosome 20 open reading frame 11	2.15	2.11	2.2		h			h
C20orf169	chromosome 20 open reading frame 169	1.85	2.04	1.65	h			h	
C20orf24	chromosome 20 open reading frame 24	2.5	2.91	2.09	h		h	h	h
C20orf36	chromosome 20 open reading frame 36	0.44	0.42	0.46			h		
C21orf25	chromosome 21 open reading frame 25	0.32	0.31	0.32				h	h
C9orf3	Chromosome 9 open reading frame 3	2.29	1.44	3.14	h	h	h		
CA12	carbonic anhydrase XII	0.33	0.18	0.47		h			
CA2	carbonic anhydrase II	2.87	0.66	5.08		hm		h	
CA9	carbonic anhydrase IX	2.39	4.14	0.63		h			
CALD1	caldesmon 1	0.11	0.14	0.09		h			
CALML3	calmodulin-like 3	2.2	0.56	3.85			h	hm	
CAPG	capping protein (actin filament), gelsolin-like	0.59	0.46	0.72				hm	hm
CASP1	caspase 1, apoptosis-related cysteine protease (interleukin 1, beta, convertase)	1.65	2.2	1.1	h				
CASP4	Caspase 4, apoptosis-related cysteine protease	1.7	2.37	1.02	h	h	h	h	h
CASR	calcium-sensing receptor	0.56	0.61	0.5				h	h
CBX3	chromobox homolog 3 (HP1 gamma homolog, Drosophila)	1.66	2.11	1.2			hm		
CBX6	chromobox homolog 6	2.17	1.87	2.46		hm			
CCL16	small inducible cytokine subfamily A (Cys-Cys), member 16	0.5	0.51	0.49	h		h		h
**CCND1**	**cyclin D1 (PRAD1: parathyroid adenomatosis 1)**	2.38	1.73	3.04		h			
CD151	CD151 antigen	11.53	13.49	9.57		h			h
**CD44**	**CD44 antigen (homing function and Indian blood group system)**	0.5	0.59	0.42					h
CD53	CD53 antigen	0.51	0.53	0.48					h

CD59	CD59 molecule, complement regulatory protein	0.4	0.36	0.44		h			
CD81	CD81 antigen (target of antiproliferative antibody 1)	0.53	0.58	0.48			h		
CD99	CD99 antigen	0.36	0.38	0.35	h		h	h	h
CDA	cytidine deaminase	0.24	0.31	0.17		h			
CDC20	CDC20 cell division cycle 20 homolog (S. cerevisiae)	1.73	2.44	1.01				hm	
CDC42EP2	Cdc42 effector protein 2	0.48	0.66	0.3	h	h	h	h	
CDC42EP4	Cdc42 effector protein 4; binder of Rho GTPases 4	1.82	1.13	2.51				hm	
**CDKN1A**	**cyclin-dependent kinase inhibitor 1A (p21, Cip1)**	0.29	0.18	0.39	h	h	h	h	h
CDKN2C	cyclin-dependent kinase inhibitor 2C (p18, inhibits CDK4)	2.01	1.31	2.71	h	h	h	h	h
CDSN	corneodesmosin	0.56	0.17	0.95	h	h	h	h	h
CDW52	CDW52 antigen (CAMPATH-1 antigen)	2.22	1.88	2.56	h		h	h	
CEACAM5	carcinoembryonic antigen-related cell adhesion molecule 5	2.62	1.08	4.16		h			h
**CEBPA**	**CCAAT/enhancer binding protein (C/EBP), alpha**	1.63	1.24	2.01	h				
CED-6	CED-6 protein	2.25	2.56	1.93		hm			
CELSR2	cadherin, EGF LAG seven-pass G-type receptor 2 (flamingo homolog, Drosophila)	1.7	1.08	2.32		h			
CENPA	centromere protein A (17kD)	1.74	2.1	1.39	h			h	h
CENTA1	centaurin, alpha 1	0.33	0.52	0.14	h		h	h	
CGN	cingulin	2.42	2.9	1.93		h			
CHCHD2	Coiled-coil-helix-coiled-coil-helix domain containing 2	1.9	2.23	1.57					h
CHK	choline kinase	2.7	2.53	2.86		h			
CHMP1.5	CHMP1.5 protein	1.64	1.04	2.25				h	h
CIA30	CGI-65 protein	2.78	2.93	2.63		h			
CIT	citron (rho-interacting, serine/threonine kinase 21)	2.42	2.28	2.57	h		h	h	
CKAP4	cytoskeleton-associated protein 4	0.43	0.38	0.49		h		h	
CKB	creatine kinase, brain	12.05	2.2	21.9		hm	h	h	
CLDN7	claudin 7	8.62	10.67	6.56	h	hm			h
CNAP1	chromosome condensation-related SMC-associated protein 1	2.28	2.36	2.2	h	h			
CNK	cytokine-inducible kinase	0.58	0.85	0.3	h	h	h		h
CNTNAP2	contactin associated protein-like 2	1.48	0.71	2.25				h	
COG3	Component of oligomeric golgi complex 3	0.28	0.35	0.2				h	h
COL12A1	collagen, type XII, alpha 1	0.22	0.19	0.26	hm			h	h
COL7A1	collagen, type VII, alpha 1	0.42	0.24	0.6	h	h	h	h	h
COL8A1	collagen, type VIII, alpha 1	0.13	0.18	0.09			h		
COPEB	core promoter element binding protein	0.22	0.25	0.19	h	h	h	h	
COTL1	Coactosin-like 1 (Dictyostelium)	0.88	1.31	0.45	h		h	h	h
CPA1	carboxypeptidase A1 (pancreatic)	0.45	0.35	0.56	h	h		h	h
CREG	cellular repressor of E1A-stimulated genes	2.6	2.03	3.16					h
CRIP1	cysteine-rich protein 1 (intestinal)	2.22	1.72	2.72		h			h
CRIP2	cysteine-rich protein 2	2.54	2.81	2.27		h			

CRSP8	cofactor required for Sp1 transcriptional activation, subunit 8 (34kD)	1.92	1.84	1.99				h	
**CSF1**	**colony stimulating factor 1 (macrophage)**	1.9	2.62	1.18	hm		hm	hm	hm
**CSF2**	**colony stimulating factor 2 (granulocyte-macrophage)**	0.23	0.26	0.2	h	h	h	h	h
CSPG2	chondroitin sulfate proteoglycan 2 (versican)	1.22	1.94	0.5	h	h	h	h	h
CTRB1	chymotrypsinogen B1	0.42	0.49	0.36		h		h	
CTSC	cathepsin C	0.47	0.61	0.34	h	h	hm	h	h
CTSH	cathepsin H	4.55	5.42	3.68		hm			
CXCL14	small inducible cytokine subfamily B (Cys-X-Cys), member 14 (BRAK)	2.11	0.27	3.95		h		h	h
CYB561	cytochrome b-561	2.18	2.59	1.77	hm	h	hm		
CYBA	cytochrome b-245, alpha polypeptide	10.55	14.32	6.78	h	h	h		h
CYBRD1	duodenal cytochrome b	0.74	0.42	1.07					h
CYP1A2	cytochrome P450, subfamily I (aromatic compound-inducible), polypeptide 2	1.56	0.88	2.23		h			
CYP26A1	cytochrome P450, subfamily XXVIA, polypeptide 1	1.78	0.64	2.93		hm	hm	h	
CYP2D6	cytochrome P450, family 2, subfamily D, polypeptide 6	0.42	0.4	0.43					h
CYP3A4	cytochrome P450, subfamily IIIA (niphedipine oxidase), polypeptide 4	0.48	0.65	0.3	h	h	hm	hm	h
CYR61	cysteine-rich, angiogenic inducer, 61	0.5	0.8	0.2	hm			hm	hm
D2S448	Melanoma associated gene	0.45	0.48	0.41					h
DATF1	death associated transcription factor 1	1.93	1.99	1.87		h			h
DAZAP2	DAZ associated protein 2	2.01	1.63	2.39	hm		hm		
DBP	D site of albumin promoter (albumin D-box) binding protein	2.35	1.57	3.13				hm	
DCAMKL1	doublecortin and CaM kinase-like 1	1.91	1.2	2.63	hm	hm	hm	hm	
DDEF1	development and differentiation enhancing factor 1	1.27	2.05	0.48		hm			
DDT	D-dopachrome tautomerase	1.82	1.61	2.02			h	h	
DDX47	DEAD (Asp-Glu-Ala-Asp) box polypeptide 47	1.75	2.15	1.35	h		h	h	
DEAF1	deformed epidermal autoregulatory factor 1 (Drosophila)	2.12	2.67	1.58		h		h	
DGKG	diacylglycerol kinase, gamma (90kD)	2.14	0.61	3.67		hm			
DISC1	disrupted in schizophrenia 1	0.17	0.18	0.15			h		
DKK3	dickkopf homolog 3 (Xenopus laevis)	0.21	0.14	0.28				h	
DLX5	distal-less homeo box 5	1.41	0.82	1.99			h	h	
DMAP1	DNA methyltransferase 1-associated protein 1	1.79	2.39	1.19	h	hm	h	h	h
DNAJB5	DnaJ (Hsp40) homolog, subfamily B, member 5	0.41	0.38	0.45	h		h	h	
DNAJC4	DnaJ (Hsp40) homolog, subfamily C, member 4	1.77	1.45	2.1	hm	hm		hm	hm
DNAJD1	DNAJ domain-containing	0.48	0.49	0.47			h		
DNM1	dynamin 1	2.5	1.62	3.39		hm			h
DOC1	downregulated in ovarian cancer 1	0.3	0.34	0.25		h	h		h
DPH2L1	Candidate tumor suppressor in ovarian cancer 2	1.97	1.84	2.1		hm		hm	hm
DPH2L2	DPH2 homolog (S. cerevisiae)	1.56	2.18	0.94	h		h		h
DPP7	dipeptidylpeptidase 7	2.59	2.56	2.61	h		h	h	h
DSG3	desmoglein 3 (pemphigus vulgaris antigen)	0.67	0.38	0.96	h			h	

DSP	desmoplakin (DPI, DPII)	0.73	0.38	1.07					h
DSTN	Destrin (actin depolymerizing factor)	0.51	0.47	0.54					h
DUSP10	dual specificity phosphatase 10	2.26	2.31	2.2	h				
DUSP22	Dual specificity phosphatase 22	0.6	0.43	0.78	h	h	h	h	h
DUSP4	dual specificity phosphatase 4	0.35	0.48	0.21		hm			
DUSP5	dual specificity phosphatase 5	0.46	0.57	0.35	h			h	h
DUSP6	dual specificity phosphatase 6	0.25	0.32	0.18	hm		hm	hm	
DXS1283E	GS2 gene	0.49	0.38	0.6				h	
EDG2	endothelial differentiation, lysophosphatidic acid G-protein-coupled receptor, 2	0.73	1.04	0.42		h			
EDG5	endothelial differentiation, sphingolipid G-protein-coupled receptor, 5	0.33	0.48	0.17			h		
EFNB1	ephrin-B1	0.25	0.11	0.39	h		h		
EFS	Embryonal Fyn-associated substrate	1.5	0.75	2.25		h		h	
EGLN1	EGL nine (C.elegans) homolog 1	0.19	0.03	0.35	h		h		
EHD1	EH-domain containing 1	0.48	0.48	0.48		h			
EIF2AK3	Eukaryotic translation initiation factor 2-alpha kinase	0.58	0.7	0.45	h		h	h	h
**ELF3**	**E74-like factor 3 (ets domain transcription factor, epithelial-specific)**	6.96	7.71	6.22					h
ELL2	ELL-RELATED RNA POLYMERASE II, ELONGATION FACTOR	0.5	0.58	0.42		h		h	h
ELTD1	EGF-TM7-latrophilin-related protein	1.77	1.41	2.13					h
EMILIN-2	extracellular glycoprotein EMILIN-2 precursor	2.34	0.86	3.82				h	
EML2	echinoderm microtubule associated protein like 2	0.58	0.38	0.77	h	h	h	h	h
EMP1	epithelial membrane protein 1	0.4	0.42	0.38					hm
ENTPD6	ectonucleoside triphosphate diphosphohydrolase 6 (putative function)	0.56	0.73	0.39		h			
EPB41	erythrocyte membrane protein band 4.1 (elliptocytosis 1, RH-linked)	0.56	0.46	0.66		hm			
EPB41L4B	EHM2 gene	2.04	1.91	2.17		hm		hm	
EPHB2	EphB2	2.02	2.44	1.61	hm	hm	hm	hm	hm
EPHB4	EphB4	0.73	0.46	1				hm	hm
EPHX1	epoxide hydrolase 1, microsomal (xenobiotic)	2.65	1.46	3.84	h	h	h	h	
EPLIN	epithelial protein lost in neoplasm beta	0.25	0.33	0.18			h		
EPPB9	B9 protein	1.6	2.11	1.1		h			
**ERBB3**	**v-erb-b2 erythroblastic leukemia viral oncogene homolog 3 (avian)**	1.74	1.46	2.02		h			
ERCC1	excision repair cross-complementing 1	0.2	0.17	0.23	h		h	h	
EREG	epiregulin	0.55	0.73	0.37		h	h	h	
ERP70	protein disulfide isomerase family A, member 4	0.41	0.49	0.33			h		
ESDN	endothelial and smooth muscle cell-derived neuropilin-like protein	0.25	0.33	0.17		h			h
ESPL1	extra spindle poles like 1 (S. cerevisiae)	1.87	1.72	2.02	h	h		h	
**ETS1**	**v-ets erythroblastosis virus E26 oncogene homolog 1 (avian)**	0.39	0.56	0.21		hm			
ETV1	ets variant gene 1	4.1	4.33	3.88	hm		h		
EVI5	ecotropic viral integration site 5	0.55	0.61	0.5		h			
EXTL1	exostoses (multiple)-like 1	0.52	0.65	0.39	h	h	h	h	h

F2RL1	coagulation factor II (thrombin) receptor-like 1	0.32	0.41	0.23		h	h		
F8A1	coagulation factor VIII-associated (intronic transcript)	2.16	2.07	2.24		h	h	h	h
FABP4	fatty acid binding protein 4, adipocyte	2.29	1.43	3.15	hm		hm	hm	
FADS2	fatty acid desaturase 2	0.16	0.17	0.14	h	h	h		
FADS3	fatty acid desaturase 3	2.69	3.98	1.4	hm	hm	hm		hm
**FASN**	**fatty acid synthase**	0.5	0.29	0.7	h				h
FASTK	FAST kinase	0.31	0.43	0.18				hm	
FBLN1	fibulin 1	1.58	1.05	2.11		hm			
FBN2	fibrillin 2 (congenital contractural arachnodactyly)	0.15	0.12	0.17	hm	hm			
FBXO2	F-box protein 2	0.53	0.63	0.42		h			
FBXW7	F-box and WD-40 domain protein 7 (archipelago homolog, Drosophila)	0.57	0.47	0.68			h	h	h
FDPS	farnesyl diphosphate synthase	0.37	0.42	0.31		h	h	h	h
FEZ1	fasciculation and elongation protein zeta 1 (zygin I)	0.2	0.1	0.3	h	h	h		
FHL1	four and a half LIM domains 1	0.13	0.19	0.08		h			
FJX1	four jointed box 1 (Drosophila)	0.52	0.65	0.4		hm		hm	
FKBP5	FK506 binding protein 5	0.26	0.23	0.29			hm	hm	
FLJ36754	Hypothetical protein FLJ36754	0.42	0.5	0.34		h			
FLOT1	flotillin 1	2.65	2.63	2.68	h	h			
FLRT2	fibronectin leucine rich transmembrane protein 2	0.4	0.4	0.41		hm			
FMO3	flavin containing monooxygenase 3	0.25	0.41	0.09			h		
**FN1**	**fibronectin 1**	0.22	0.42	0.01	h	h	h	h	h
FNTA	farnesyltransferase, CAAX box, alpha	1.63	0.9	2.35		h		h	
FOLR3	folate receptor 3 (gamma)	0.24	0.28	0.2	h	h		h	h
FOSL1	FOS-like antigen 1	0.43	0.37	0.49					h
FOXM1	forkhead box M1	2.52	2.47	2.57	hm	hm			
FST	follistatin	0.14	0.11	0.17		h			
FSTL3	follistatin-like 3 (secreted glycoprotein)	0.24	0.23	0.25	hm	hm			
FUT8	fucosyltransferase 8 (alpha (1,6) fucosyltransferase)	1.53	2.01	1.05	hm			hm	
FXYD3	FXYD domain-containing ion transport regulator 3	1.44	0.75	2.14		hm		h	
FY	Duffy blood group	0.58	0.69	0.48	h	h	hm	h	h
FYB	FYN binding protein (FYB-120/130)	4.23	5.3	3.16	hm		hm		h
G0S2	putative lymphocyte G0/G1 switch gene	0.56	0.83	0.29			h		
GAA	glucosidase, alpha; acid (Pompe disease, glycogen storage disease type II)	3.5	2.64	4.35	h	h		h	
GABARAPL1	GABA(A) receptor-associated protein like 1	2.31	2.25	2.37		hm			
GABRA6	gamma-aminobutyric acid (GABA) A receptor, alpha 6	0.57	0.72	0.43			h		
GABRE	gamma-aminobutyric acid (GABA) A receptor, epsilon	1.07	0.45	1.69					hm
GAGE8	G antigen 2	1.87	1.18	2.57	h	h			
GAL	galanin	5.1	3.04	7.16		h	h	h	
GAS1	growth arrest-specific 1	1.41	0.48	2.34				h	h

GATA2	GATA binding protein 2	2.48	2.88	2.07		hm			
GBAS	glioblastoma amplified sequence	1.87	1.69	2.04	h		h	h	h
GCA	grancalcin, EF-hand calcium binding protein	2.11	1.6	2.61	h	h	h		
GCG	glucagon	0.49	0.63	0.35	hm	hm	hm		
GDF5	growth differentiation factor 5 (cartilage-derived morphogenetic protein-1)	1.87	1.2	2.54		hm		hm	
GFRA3	GDNF family receptor alpha 3	0.47	0.59	0.34		h			
GHRH	growth hormone releasing hormone	0.43	0.39	0.47		h		h	h
GJA1	gap junction protein, alpha 1, 43kD (connexin 43)	0.94	0.2	1.68					hm
GJA5	gap junction protein, alpha 5, 40kD (connexin 40)	0.38	0.38	0.38	hm		hm	hm	
GJB2	gap junction protein, beta 2, 26kD (connexin 26)	0.56	0.42	0.71	h	h			
GJB3	gap junction protein, beta 3, 31kD (connexin 31)	0.44	0.44	0.44	h	h	h	h	h
GJB5	gap junction protein, beta 5 (connexin 31.1)	0.4	0.32	0.48	h				
GMPPB	GDP-mannose pyrophosphorylase B	0.52	0.58	0.45	hm	h	hm	hm	hm
GNA15	G protein, alpha 15 (Gq class)	0.36	0.35	0.37	h	h	h	h	
GNAI2	G protein, alpha inhibiting activity polypeptide 2	0.47	0.49	0.45	h				h
GNB1	G protein, beta polypeptide 1	0.52	0.43	0.61	hm	hm	hm	hm	
GNB2	G protein, beta polypeptide 2	0.65	0.8	0.5				h	h
GNB4	G protein beta subunit 4	0.52	0.6	0.44		h			
GNG12	G protein gamma-12 subunit	0.38	0.43	0.32		h			
GOLPH3	golgi phosphoprotein 3 (coat-protein)	1.92	2.06	1.78		hm			
GPC1	glypican 1	0.47	0.36	0.59		hm		hm	
GPR109B	G protein-coupled receptor 109B	3.12	2.94	3.3	h		h		
GPRC5B	G protein-coupled receptor, family C, group 1, member B	1.66	2.02	1.31				h	
GPX2	glutathione peroxidase 2 (gastrointestinal)	6.46	2.55	10.37				hm	
GPX4	glutathione peroxidase 4 (phospholipid hydroperoxidase)	1.76	1.31	2.2		h		h	
GSTM2	glutathione S-transferase M2 (muscle)	1.15	0.34	1.96		h			
GTF2A1	general transcription factor IIA, 1 (37kD and 19kD subunits)	0.43	0.34	0.52				h	
GTF2H2	general transcription factor IIH, polypeptide 2 (44kD subunit)	0.69	0.98	0.4	h	h	h		
H1F0	H1 histone family, member 0	1.49	0.96	2.02	hm	hm	hm	hm	
HAP1	huntingtin-associated protein 1 (neuroan 1)	1.89	1.48	2.3		h			
HBE1	hemoglobin, epsilon 1	1.84	1.19	2.49		h	h	h	h
HCA112	hepatocellular carcinoma-associated antigen 112	1.51	1.02	2	h	h	h	h	h
HCG4P6	HLA complex group 4 pseudogene 6	0.58	0.47	0.69	h	h	h	h	h
HERC3	hect domain and RLD 3	0.3	0.37	0.23				h	h
HIBADH	3-hydroxyisobutyrate dehydrogenase	2.04	2.29	1.79				h	
HIST1H2BD	H2B histone family, member B	4.49	1.64	7.34	h	h	h	h	h
HIST1H4B	H4 histone family, member I	1.96	2.21	1.7	h				
HIST1H4C	H4 histone family, member G	2.73	3.25	2.21				h	h
HIST2H2BE	H2B histone family, member Q	1.71	0.98	2.43	h	h	h	h	h

HLA-C	major histocompatibility complex, class I, C	2.37	1.2	3.53	h	h	h	h	h
HLA-DMA	major histocompatibility complex, class II, DM alpha	2.8	1.38	4.22	h				
HLA-DMB	major histocompatibility complex, class II, DM beta	1.44	0.61	2.27	h	h	h	h	h
HLA-E	major histocompatibility complex, class I, E	1.64	0.64	2.64		h			
**HLA-F**	**major histocompatibility complex, class I, F**	1.7	0.86	2.54		h		h	
**HLA-G**	**HLA-G histocompatibility antigen, class I, G**	1.79	0.88	2.7					h
HLF	hepatic leukemia factor	0.5	0.42	0.58		hm		hm	hm
HMGCR	3-hydroxy-3-methylglutaryl-Coenzyme A reductase	0.4	0.47	0.33					h
HOXA13	homeo box A13	2.01	1.69	2.33	h	h		h	h
HOXB2	homeo box B2	0.71	0.47	0.95					h
HOXB9	homeo box B9	2.18	2.67	1.7	hm	hm			
HOXD9	homeo box D9	2.41	1.83	2.99	h		h	h	
HR	hairless (mouse) homolog	0.23	0.19	0.27		hm			
HSPA4L	Heat shock 70kDa protein 4-like	0.61	0.4	0.82			h	h	
HSPB8	Heat shock 22kDa protein 8	2.1	1.49	2.72	h			h	
HSUP1	Similar to RPE-spondin	2.52	2.74	2.3	h	h		h	h
**HSXIAPAF1**	**XIAP associated factor-1**	1.61	0.96	2.26				h	
HT008	uncharacterized hypothalamus protein HT008	0.48	0.46	0.49	h	hm			
HTATIP	HIV-1 Tat interactive protein, 60 kD	1.64	2.01	1.28	hm		hm		
**ICAM1**	**intercellular adhesion molecule 1 (CD54)**	2.39	3.38	1.4	hm	h	hm		
ICK	Intestinal cell (MAK-like) kinase	1.98	1.09	2.87		h		h	
ID2	inhibitor of DNA binding 2, dominant negative helix-loop-helix protein	2.36	0.86	3.86				hm	h
IDI1	isopentenyl-diphosphate delta isomerase	0.33	0.31	0.35				h	
IFITM1	interferon induced transmembrane protein 1 (9-27)	1.39	0.3	2.48				h	h
**IGF2**	**insulin-like growth factor 2 (somatomedin A)**	2.86	3.29	2.43		h			
**IGFBP2**	**insulin-like growth factor binding protein 2 (36kD)**	12.77	1.02	24.52	h	h		h	
IGFBP3	insulin-like growth factor binding protein 3	33.12	15.91	50.34					h
IGFBP6	insulin-like growth factor binding protein 6	3.64	2.51	4.77	hm	hm	hm	hm	
**IKBKE**	**IKK-related kinase epsilon; inducible IkappaB kinase**	1.74	2.14	1.35	h		h	h	h
**IL1A**	**interleukin 1, alpha**	0.34	0.42	0.26	hm			hm	hm
**IL1B**	**interleukin 1, beta**	0.66	0.94	0.37	hm	h	hm	hm	
IL1R2	interleukin 1 receptor, type II	0.15	0.13	0.17	hm		hm	hm	
**IL1RN**	**interleukin 1 receptor antagonist**	0.66	0.35	0.97					h
IL22RA1	interleukin 22 receptor	3.13	2.3	3.96	hm			hm	hm
**IL2RA**	**interleukin 2 receptor, alpha**	1.83	1.19	2.48	hm	hm	hm	hm	hm
**IL32**	**interleukin 32**	3.57	2.94	4.2	h	h	h	h	h
IL4R	interleukin 4 receptor	0.44	0.59	0.3	h	h	h	h	
**IL6**	**interleukin 6 (interferon, beta 2)**	4.18	6.34	2.01	hm	hm	hm		hm
IL6ST	interleukin 6 signal transducer (gp130, oncostatin M receptor)	0.65	0.82	0.47		h			

**IL8**	**interleukin 8**	5.21	9.3	1.13	h		h	h	h
IMP-1	IGF-II mRNA-binding protein 1	1.87	2.45	1.3	hm		hm	hm	hm
IMPA2	inositol(myo)-1(or 4)-monophosphatase 2	1.95	0.72	3.17				h	h
INA	internexin neuronal intermediate filament protein, alpha	1.56	0.75	2.37			h		
INHBA	inhibin, beta A (activin A, activin AB alpha polypeptide)	0.33	0.36	0.3	hm		hm	hm	
IPO11	Importin 11	0.68	0.87	0.49		h			
IQGAP2	IQ motif containing GTPase activating protein 2	0.5	0.44	0.56					h
**IRF4**	**interferon regulatory factor 4**	0.5	0.42	0.58	h			h	h
IRF5	interferon regulatory factor 5	0.5	0.6	0.4		h			h
IRS1	insulin receptor substrate 1	1.75	0.94	2.56		hm	hm		
ISYNA1	myo-inositol 1-phosphate synthase A1	2.2	2.17	2.23		h		hm	hm
ITGA2	integrin, alpha 2 (CD49B, alpha 2 subunit of VLA-2 receptor)	0.44	0.44	0.45	h	h			
ITGA5	integrin, alpha 5 (fibronectin receptor, alpha polypeptide)	0.26	0.29	0.23	hm	hm	hm	hm	
**ITGA6**	**integrin, alpha 6**	0.43	0.3	0.56		h			
ITGB4	integrin, beta 4	0.41	0.36	0.46	h	h		h	
ITPKA	inositol 1,4,5-trisphosphate 3-kinase A	2.48	2.13	2.83	h	h		h	h
IVL	involucrin	0.72	0.43	1.02		h			
JM4	JM4 protein	0.54	0.49	0.59	hm		hm	hm	hm
JRKL	jerky homolog-like (mouse)	1.67	2.37	0.97	h	h	h	h	h
**JUND**	**jun D proto-oncogene**	0.51	0.4	0.62		h			
KCND2	potassium voltage-gated channel, Shal-related subfamily, member 2	1.27	0.48	2.05		hm			
KCNK6	potassium channel, subfamily K, member 6 (TWIK-2)	0.45	0.48	0.43	h	h	h		
**KCNN4**	**K intermediate/small conductance Ca-activated channel, subfamily N, member 4**	4.16	6.74	1.59		hm			
KIAA0420	KIAA0420 gene product	0.59	0.5	0.68				h	h
KIAA0738	KIAA0738 protein	1.69	2.55	0.83			h		
KIAA0992	palladin	0.41	0.42	0.39	h		h	h	h
KIAA1102	KIAA1102 protein	5.14	4.47	5.81	h	h			h
KIF23	kinesin-like 5 (mitotic kinesin-like protein 1)	1.73	2.05	1.41	h			h	h
KIF2C	kinesin-like 6 (mitotic centromere-associated kinesin)	3.14	4.18	2.1	h				
KIP2	DNA-dependent protein kinase catalytic subunit-interacting protein 2	0.49	0.55	0.43		hm			
KIT	v-kit Hardy-Zuckerman 4 feline sarcoma viral oncogene homolog	0.48	0.4	0.56				hm	
KLF7	Kruppel-like factor 7 (ubiquitous)	0.45	0.51	0.4		h			
KLK10	kallikrein 10	1.58	0.37	2.78		h	h	h	
KLK11	kallikrein 11	2.2	0.67	3.74					h
KLK5	kallikrein 5	0.71	0.34	1.08	h	h			
KRT1	keratin 1 (epidermolytic hyperkeratosis)	2.7	1.58	3.83				hm	
KRT14	keratin 14 (epidermolysis bullosa simplex, Dowling-Meara, Koebner)	0.47	0.21	0.72	hm				
KRT16	keratin 16 (focal non-epidermolytic palmoplantar keratoderma)	0.37	0.13	0.61		h			
KRT19	keratin 19	4.81	4.31	5.31			h	h	

KRT4	keratin 4	5.4	0.79	10.02		hm			
**KRT6B**	**keratin 6B**	0.3	0.12	0.48	h			h	
KRT8	keratin 8	1.98	3.06	0.91	h	h	h		
KRTAP2-4	keratin associated protein 2-4	1.7	2.28	1.12	h		h		
LAMA3	laminin, alpha 3	0.17	0.12	0.22	h				
LAMB3	laminin, beta 3	0.39	0.31	0.47	hm	hm	hm	hm	
LAMC2	laminin, gamma 2	0.31	0.23	0.39		h			
LAMP3	lysosomal-associated membrane protein 3	3.09	2.27	3.91	h		h		h
LARS2	leucyl-tRNA synthetase, mitochondrial	0.65	0.81	0.49					h
**LBP**	**lipopolysaccharide binding protein**	1.52	0.92	2.13				h	h
LBP-32	LBP protein 32	2.27	1.96	2.58	h		h		h
LCN2	lipocalin 2 (oncogene 24p3)	2.97	3.3	2.65	h		h		h
LDLR	low density lipoprotein receptor (familial hypercholesterolemia)	0.46	0.52	0.4					h
LGALS7	lectin, galactoside-binding, soluble, 7 (galectin 7)	0.62	0.47	0.77				h	h
**LGN**	**LGN protein**	0.54	0.49	0.59					h
LILRB3	leukocyte immunoglobulin-like receptor, subfamily B, member 3	0.5	0.38	0.62		h		h	h
LIPG	lipase, endothelial	0.19	0.2	0.18		h	h		
LIX1L	Lix1 homolog (mouse) like	0.3	0.4	0.2		h			
LMAN1	lectin, mannose-binding, 1	0.66	0.82	0.49		hm			
LMO2	LIM domain only 2 (rhombotin-like 1)	1.51	0.68	2.34		h	h	h	h
LMO7	LIM domain only 7	3.47	2.69	4.26	h				
LOC440395	LOC440395	0.55	0.62	0.49		h			
LOC51760	B/K protein	1.68	2.14	1.22	h		h	h	
LOC83690	CocoaCrisp	5.04	2.42	7.65		h			
LOXL2	lysyl oxidase-like 2	1.02	1.91	0.14	h	h	h	h	h
LPIN1	lipin 1	0.23	0.2	0.26			h		
LRP8	low density lipoprotein receptor-related protein 8, apolipoprotein e receptor	0.71	0.96	0.46		h		h	
LRRFIP1	leucine rich repeat (in FLII) interacting protein 1	0.6	0.71	0.48					h
LTBP2	latent transforming growth factor beta binding protein 2	0.4	0.55	0.24	h	h	h	hm	h
MAFB	v-maf musculoaponeurotic fibrosarcoma oncogene homolog B (avian)	2.09	0.99	3.19				hm	
MAGEA11	melanoma antigen, family A, 11	2.2	1.86	2.55	h			h	
MAGEA5	melanoma antigen, family A, 5	2.05	1.46	2.64		h			
MAGEB2	melanoma antigen, family B, 2	2.5	4	1		h			h
MAGEF1	MAGEF1 protein	2.94	1.8	4.08	h		h	h	
MAGP2	Microfibril-associated glycoprotein-2	0.27	0.3	0.23	hm				
MALT1	mucosa associated lymphoid tissue lymphoma translocation gene 1	0.34	0.35	0.33		h			
MAN1A1	mannosidase, alpha, class 1A, member 1	0.29	0.2	0.39		hm			
MAP17	epithelial protein up-regulated in carcinoma, membrane associated protein 17	1.76	1	2.52	h	h	h	h	h
MAP4	microtubule-associated protein 4	0.51	0.6	0.42		h			

MAP4K4	mitogen-activated protein kinase kinase kinase kinase 4	0.59	0.77	0.42				h	
MAPKAPK3	mitogen-activated protein kinase-activated protein kinase 3	0.3	0.38	0.21				h	
MARK4	MAP/microtubule affinity-regulating kinase like 1	0.73	0.47	1	h	h	h	h	
MATN2	matrilin 2	4.11	1.57	6.64		h			
MBD1	methyl-CpG binding domain protein 1	0.37	0.3	0.44		h		h	h
MCAM	melanoma cell adhesion molecule	0.21	0.39	0.03	h	h	h		h
MCCC1	methylcrotonoyl-Coenzyme A carboxylase 1 (alpha)	3.02	2.17	3.88	h	h	h	h	
MCL1	myeloid cell leukemia sequence 1 (BCL2-related)	0.61	0.76	0.47			hm	hm	
MDFI	MyoD family inhibitor	0.46	0.39	0.52	h	h			h
MDK	midkine (neurite growth-promoting factor 2)	2.53	2.35	2.72		hm		h	
MDS030	uncharacterized hematopoietic stem/progenitor cells protein MDS030	0.41	0.4	0.42	h		h	h	h
MEIS2	Meis1, myeloid ecotropic viral integration site 1 homolog 2 (mouse)	2.24	1.81	2.66					h
MGLL	monoglyceride lipase	0.39	0.44	0.35	h	hm			
MLL	myeloid/lymphoid or mixed-lineage leukemia (trithorax homolog, Drosophila)	0.36	0.41	0.31		h		h	h
MLPH	melanophilin	2.4	3.46	1.33	h	h	h	h	h
**MMP13**	**matrix metalloproteinase 13 (collagenase 3)**	2.66	1.85	3.47				h	
MO25	MO25 protein	0.42	0.52	0.32		h			
MPHOSPH6	M-phase phosphoprotein 6	0.59	0.49	0.68		h			
MPHOSPH9	M-phase phosphoprotein 9	1.63	1.23	2.02			h	h	
MPP1	membrane protein, palmitoylated 1 (55kD)	1.96	1.12	2.79			h	h	h
MRPL21	mitochondrial ribosomal protein L21	2.83	1.95	3.71	h		h	h	h
MRPS14	mitochondrial ribosomal protein S14?	1.73	1.31	2.16		h			
MSLN	mesothelin	2.06	0.79	3.33				h	
**MSX1**	**msh homeo box homolog 1 (Drosophila)**	2.46	2.97	1.95	h	h	h	h	h
MT1E	metallothionein 1E (functional)	0.47	0.63	0.31	h	h	h		h
MT1F	Metallothionein 1F (functional)	0.39	0.54	0.23	h	h	h	h	
MT1G	metallothionein 1G	0.46	0.63	0.3		h			
MT1H	metallothionein 1H	0.45	0.58	0.33	h		h	h	
MT1L	Metallothionein 1L	0.35	0.49	0.21	h	h	h	h	
MT2A	metallothionein 2A	0.31	0.47	0.15	h	h	h	h	
MTMR9	myotubularin related protein 8	0.44	0.42	0.46			h	h	
MUC1	mucin 1, transmembrane	2.26	1.74	2.78	hm		hm		
MUC4	mucin 4, tracheobronchial	3.52	5.07	1.96		h			h
MVD	mevalonate (diphospho) decarboxylase	0.39	0.43	0.35	h	h		h	h
MX1	myxovirus (influenza virus) resistance 1, interferon-inducible protein p78 (mouse)	0.57	0.18	0.95					h
MYBL2	v-myb myeloblastosis viral oncogene homolog (avian)-like 2	2.71	3.39	2.03	h	h	h		
MYH11	myosin, heavy polypeptide 11, smooth muscle	0.54	0.74	0.35					h
MYH3	myosin, heavy polypeptide 3, skeletal muscle, embryonic	0.47	0.61	0.33			h		
MYO1E	myosin IE	0.44	0.53	0.35	hm		hm	hm	

MYO5B	myosin VB	2.49	2.01	2.97			h	h	
MYST3	MYST histone acetyltransferase (monocytic leukemia) 3	1.77	0.92	2.61				h	
NALP1	death effector filament-forming Ced-4-like apoptosis protein	0.48	0.4	0.57	h	h		h	h
NALP2	NALP2 protein	0.49	0.49	0.49		h			
NDRG1	N-myc downstream regulated gene 1	0.44	0.38	0.5			h		
NDUFB9	NADH dehydrogenase (ubiquinone) 1 beta subcomplex, 9 (22kD, B22)	2.03	2.48	1.58	h		h		
NDUFV1	NADH dehydrogenase (ubiquinone) flavoprotein 1 (51kD)	1.98	1.93	2.03	h		h	h	
NEDD4L	neural precursor cell expressed, developmentally down-regulated 4-like	0.35	0.21	0.5	h		h		
NEK2	NIMA (never in mitosis gene a)-related kinase 2	2.36	2.32	2.39					h
NEO1	neogenin (chicken) homolog 1	1.65	1.03	2.27	hm	hm	hm	hm	
NEUROD2	neurogenic differentiation 2	0.5	0.34	0.66		hm			h
NFE2L2	nuclear factor (erythroid-derived 2)-like 2	1.39	0.74	2.03		hm	h	h	
NID67	putative small membrane protein NID67	0.39	0.47	0.32		h	h	h	h
NKX3-1	NK3 transcription factor homolog A (Drosophila)	0.59	0.68	0.5		h			
NMB	neuromedin B	0.61	0.32	0.91	h				
NME4	non-metastatic cells 4, protein expressed in	0.46	0.63	0.3	h	h	h	h	h
NMES1	normal mucosa of esophagus specific 1	1.78	1.29	2.28			h		
**NOS2A**	**nitric oxide synthase 2A (inducible, hepatocytes)**	0.5	0.42	0.58				h	h
NOT56L	Not56 (D. melanogaster)-like protein	1.83	1.65	2.01			hm	hm	
NP	nucleoside phosphorylase	0.54	0.7	0.37		h	h	h	h
NP25	neuronal protein	0.3	0.46	0.14	hm	hm	hm	hm	
NPR2L	homologous to yeast nitrogen permease (candidate tumor suppressor)	0.64	0.82	0.46					h
**NQO1**	**NAD(P)H dehydrogenase, quinone 1**	2.63	2.3	2.96					h
**NR4A1**	**nuclear receptor subfamily 4, group A, member 1**	0.26	0.5	0.01	h	hm	h	h	h
NRG2	neuregulin 2	0.48	0.35	0.61				h	h
NT5E	5' nucleotidase (CD73)	0.15	0.14	0.16			h		
NTRK2	neurotrophic tyrosine kinase, receptor, type 2	4.67	0.67	8.67				h	
NUP155	nucleoporin 155kD	1.74	2.14	1.33	h			h	h
**OAS2**	**2'-5'-oligoadenylate synthetase 2, 69/71kDa**	1.34	0.26	2.43		h		h	
OAT	ornithine aminotransferase (gyrate atrophy)	0.49	0.51	0.47	h				
OGFR	opioid growth factor receptor	1.85	1.55	2.16	h	h		h	
OLFM1	olfactomedin 1	2.12	1.03	3.2		h	h	h	
OSBPL10	oxysterol binding protein-like 10	0.25	0.33	0.18	h		h	h	h
OSR1	oxidative-stress responsive 1	0.46	0.54	0.37		h			
P5CR2	pyrroline 5-carboxylate reductase isoform	1.65	1.26	2.03		h			
P8	p8 protein (candidate of metastasis 1)	0.51	0.31	0.71	h		h	h	
PALM	Paralemmin	1.86	2.05	1.67		hm			
PALMD	palmdelphin	1.61	1.02	2.2				hm	
PANX1	pannexin 1	0.52	0.57	0.48	h		h	h	h

PAPSS1	3'-phosphoadenosine 5'-phosphosulfate synthase 1	0.46	0.45	0.47		hm		hm	
**PDE7A**	**phosphodiesterase 7A**	0.71	0.96	0.46		h			
PDIR	for protein disulfide isomerase-related	0.63	0.81	0.45	h	h	h	h	h
PDK1	pyruvate dehydrogenase kinase, isoenzyme 1	1.34	2.03	0.65					h
PDK2	pyruvate dehydrogenase kinase, isoenzyme 2	2.31	1.78	2.84	hm	hm	hm	h	
PEA15	phosphoprotein enriched in astrocytes 15	0.54	0.64	0.44	hm	hm	hm	hm	hm
PEG3	Paternally expressed 3	0.55	0.44	0.66	h		h		
PELI2	pellino homolog 2 (Drosophila)	0.42	0.37	0.47				h	h
PFKP	phosphofructokinase, platelet	0.72	1.07	0.38		h	h		
PGF	placental growth factor, vascular endothelial growth factor-related protein	0.15	0.14	0.16		hm			
PGM1	phosphoglucomutase 1	1.01	1.58	0.45		h			
PHLDA1	pleckstrin homology-like domain, family A, member 1	0.33	0.38	0.28	hm	h	hm	hm	hm
PIK3R3	phosphoinositide-3-kinase, regulatory subunit, polypeptide 3 (p55, gamma)	2.32	1.86	2.77					h
**PIM1**	**pim-1 oncogene**	2.05	2.25	1.84	hm	hm			
PLA2G4B	phospholipase A2, group IVB (cytosolic)	1.7	1.05	2.34			h		
PLAT	plasminogen activator, tissue	1.65	2.13	1.17	hm		hm	h	h
**PLAU**	**plasminogen activator, urokinase**	0.31	0.36	0.27			h		
**PLCD1**	**Phospholipase C, delta 1**	0.3	0.16	0.45		h		hm	hm
PLCG2	phospholipase C, gamma 2 (phosphatidylinositol-specific)	3.63	1.6	5.66	h	h	h	h	h
PLEK2	pleckstrin 2 (mouse) homolog	0.29	0.35	0.23	h	hm	h		
PLEKHF2	phafin 2	1.67	1.02	2.33			h		
PLK	polo-like kinase (Drosophila)	2.1	2.68	1.51				h	
PLXNB1	plexin B1	0.6	0.47	0.72				h	
PMP22	peripheral myelin protein 22	0.37	0.38	0.36		h		h	h
PMS2	PMS2 postmeiotic segregation increased 2 (S. cerevisiae)	2.05	1.78	2.31				h	h
POLE3	polymerase (DNA directed), epsilon 3 (p17 subunit)	2.01	2.09	1.92	hm				
POLH	polymerase (DNA directed), eta	0.34	0.34	0.35				h	
POLR2L	polymerase (RNA) II (DNA directed) polypeptide L (7.6kD)	0.54	0.68	0.4	h	h		h	
**POMC**	**proopiomelanocortin**	0.54	0.72	0.37		h			
POMT1	protein-O-mannosyltransferase 1	0.66	0.84	0.48		h			h
PORIMIN	pro-oncosis receptor inducing membrane injury gene	2.8	4.33	1.27	h	h			h
POU3F4	POU domain, class 3, transcription factor 4	0.24	0.23	0.25	hm	hm		hm	
PPAP2C	phosphatidic acid phosphatase type 2C	4.36	4	4.71	h		h		h
PPARBP	PPAR binding protein	0.21	0.28	0.15	hm	hm	hm		h
PPARG	peroxisome proliferative activated receptor, gamma	2.22	3.22	1.21		h		h	
PPFIA1	PTPRF interacting protein alpha 1	2.05	1.43	2.68		h		h	
PPM2C	protein phosphatase 2C, magnesium-dependent, catalytic subunit	0.71	0.94	0.48	h				
PPP2R5B	protein phosphatase 2, regulatory subunit B (B56), beta isoform	0.47	0.41	0.54		hm			h
PPP3CC	protein phosphatase 3 (formerly 2B), catalytic subunit, gamma isoform	0.48	0.46	0.49		h			

PRAME	preferentially expressed antigen in melanoma	2.9	4.25	1.55		h			
**PRG1**	**proteoglycan 1, secretory granule**	0.34	0.38	0.31		h		h	
PRG3	proteoglycan 3	1.67	0.75	2.59				h	
PRKCABP	protein kinase C, alpha binding protein	0.78	0.36	1.2				h	h
PRKCQ	protein kinase C, theta	0.47	0.38	0.57				h	
PRKX	protein kinase, X-linked	0.48	0.28	0.67		h			
PRNP	prion protein (p27-30)	0.39	0.31	0.48		h			
PRODH	proline oxidase homolog	2.51	0.84	4.19		h	h	h	
PROSC	proline synthetase co-transcribed homolog (bacterial)	1.49	0.92	2.06				h	
PRSS3	protease, serine, 4 (trypsin 4, brain)	0.32	0.2	0.44			h		
PSG1	pregnancy specific beta-1-glycoprotein 1	0.67	0.96	0.38	h		h	h	
PSG5	pregnancy specific beta-1-glycoprotein 5	0.55	0.64	0.45				h	
PSMB10	proteasome (prosome, macropain) subunit, beta type, 10	1.82	2.12	1.52			h		
PSORS1C2	Psoriasis susceptibility 1 candidate 2	2.29	1.36	3.21	h		h		h
PTGES	prostaglandin E synthase	2.7	2.93	2.47	h	h	h	h	
PTGFRN	Prostaglandin F2 receptor negative regulator	0.62	0.5	0.73	h	h	h	h	
**PTGS1**	**prostaglandin-endoperoxide synthase 1**	2.89	1.84	3.94	hm	h	hm	hm	hm
**PTGS2**	**prostaglandin-endoperoxide synthase 2**	0.08	0.07	0.1		h			h
**PTHLH**	**parathyroid hormone-like hormone**	0.6	0.73	0.47				h	
PTHR2	parathyroid hormone receptor 2	0.39	0.36	0.43	h	h	h	h	
PTK6	PTK6 protein tyrosine kinase 6	0.48	0.43	0.52		h		h	
PTPN7	protein tyrosine phosphatase, non-receptor type 7	1.58	0.69	2.46	hm	hm		hm	hm
PTPRA	protein tyrosine phosphatase, receptor type, A	2.69	3.36	2.02		h			hm
PTPRJ	protein tyrosine phosphatase, receptor type, J	2.19	2.96	1.43		h			
PTPRN2	protein tyrosine phosphatase, receptor type, N polypeptide 2	1.46	0.9	2.01		h			h
PVALB	parvalbumin	0.55	0.6	0.5	h		h	h	h
PVRL1	poliovirus receptor-related 1 (herpesvirus entry mediator C; nectin)	4.4	0.98	7.81				h	
RAB17	RAB17, member RAS oncogene family	1.9	2.7	1.11					h
RAB22A	RAB22A, member RAS oncogene family	2.37	1.72	3.02		h			h
RABGAP1	RAB GTPase activating protein 1	1.76	1.34	2.19	h	h	h		
RAC2	Ras-related C3 botulinum toxin substrate 2	0.49	0.65	0.34	h	hm		h	h
RAD1	RAD1 homolog (S. pombe)	1.81	2.25	1.37	h	h			h
RAD54L	RAD54-like (S. cerevisiae)	1.53	2	1.05	h			h	h
RALGDS	ral guanine nucleotide dissociation stimulator	1.99	1.41	2.58				h	
RAMP2	receptor (calcitonin) activity modifying protein 2	1.65	2.07	1.24	h		h	h	h
RAP1GDS1	RAP1, GTP-GDP dissociation stimulator 1	0.46	0.45	0.46			h	h	
RARRES3	retinoic acid receptor responder (tazarotene induced) 3	3.31	2.43	4.18		h			
RASA1	RAS p21 protein activator (GTPase activating protein) 1	0.56	0.69	0.44		h			
RASD1	RAS, dexamethasone-induced 1	0.18	0.21	0.16		h		h	

**RBBP4**	**retinoblastoma binding protein 4**	1.56	2.04	1.07	h	h	h	hm	h
RBM13	RNA binding motif protein 13	1.88	1.09	2.68		h		h	
RBPMS	RNA-binding protein gene with multiple splicing	0.37	0.44	0.3	h				
RECQL4	RecQ protein-like 4	2.21	2.43	1.98				h	
**REL**	**v-rel reticuloendotheliosis viral oncogene homolog (avian)**	1.75	1.18	2.33	h	h	hm	h	h
RGS2	regulator of G-protein signalling 2, 24kD	2.44	0.76	4.11		h	h		
RHOA	ras homolog gene family, member A	0.47	0.52	0.42	h				
RHOB	ras homolog gene family, member B	0.51	0.43	0.59		hm			
RHOC	ras homolog gene family, member C	0.45	0.53	0.38	h		h	h	
RNF139	patched related protein translocated in renal cancer	1.61	2.02	1.21				h	
RNPC1	RNA-binding region (RNP1, RRM) containing 1	2.52	2.86	2.18	h	h	h		
RNPS1	RNA binding protein S1, serine-rich domain	2.54	2.78	2.31		hm		h	h
RPL22L1	ribosomal protein L22-like 1	0.65	1	0.3		h			
RPP25	Ribonuclease P 25kDa subunit	1.88	1.62	2.14	h	h	h	h	
RPS6KA1	ribosomal protein S6 kinase, 90kD, polypeptide 1	0.51	0.4	0.63	h		h	h	h
RPS6KA2	ribosomal protein S6 kinase, 90kD, polypeptide 2	0.5	0.4	0.6	h	h	h	h	h
RREB1	ras responsive element binding protein 1	1.54	0.81	2.27		h		h	h
RSU1	Ras suppressor protein 1	0.45	0.44	0.46	h	h	h		
RYR1	ryanodine receptor 1 (skeletal)	1.84	1.33	2.35			h	h	h
S100A2	S100 calcium binding protein A2	0.39	0.38	0.4	h	h	h	h	h
S100A3	S100 calcium binding protein A3	0.63	0.46	0.8	h		h	h	
S100A4	S100 calcium binding protein A4	0.53	0.39	0.67		h		h	
**S100A6**	**S100 calcium binding protein A6 (calcyclin)**	0.21	0.15	0.27				h	h
S100A9	S100 calcium binding protein A9 (calgranulin B)	0.87	0.36	1.39	hm			hm	
**SAA1**	**serum amyloid A1**	1.64	1.25	2.03	h		h	h	h
SACS	spastic ataxia of Charlevoix-Saguenay (sacsin)	0.34	0.46	0.22		h	h		
SART2	squamous cell carcinoma antigen recognized by T cell	0.51	0.49	0.52				h	
SAT	spermidine/spermine N1-acetyltransferase	0.27	0.29	0.26	h	h	h	h	h
SCAM-1	vinexin beta (SH3-containing adaptor molecule-1)	0.47	0.58	0.37			h		
SCD	stearoyl-CoA desaturase (delta-9-desaturase)	0.22	0.28	0.17	h	h	h	h	
SCYL1	N-terminal kinase-like	1.6	2.09	1.11	hm		hm	hm	hm
SDR1	short-chain dehydrogenase/reductase 1	4.71	5.27	4.14	h	h			h
SECTM1	secreted and transmembrane 1	2.51	1.63	3.39					h
SENP2	sentrin-specific protease	2.06	1.82	2.3				h	h
SEPP1	selenoprotein P, plasma, 1	0.25	0.15	0.35				h	
SERPINB1	serine (or cysteine) proteinase inhibitor, clade B (ovalbumin), member 1	1.9	2.14	1.66	h		h	h	h
SERPINB7	serine (or cysteine) proteinase inhibitor, clade B (ovalbumin), member 7	0.11	0.09	0.13	h		h	h	h
**SERPINE1**	**serine (or cysteine) proteinase inhibitor, clade E**	0.32	0.46	0.18			hm		
**SERPINE2**	**Serine (or cysteine) proteinase inhibitor, clade E, member 2**	0.07	0.04	0.1		h	h		h

SERPINF1	serine (or cysteine) proteinase inhibitor, clade F, member 1	4.24	1.65	6.83		h			
SERPING1	serine (or cysteine) proteinase inhibitor, clade G (C1 inhibitor), member 1	0.31	0.35	0.28	h	h	h	hm	hm
SERPINI1	serine (or cysteine) proteinase inhibitor, clade I (neuroserpin), member 1	3.23	0.95	5.51					hm
SEZ6L2	Seizure related 6 homolog (mouse)-like 2	3.23	3.03	3.44	hm	hm		hm	
SFN	stratifin	0.4	0.38	0.42	h		h	h	h
SFRP1	secreted frizzled-related protein 1	0.08	0.09	0.07			h		
SFRS11	splicing factor, arginine/serine-rich 11	0.45	0.2	0.7	h				
SH2D2A	SH2 domain protein 2A	0.4	0.36	0.45	h			h	
**SH3BGRL**	**SH3 domain binding glutamic acid-rich protein like**	0.46	0.51	0.42					hm
SH3KBP1	SH3-domain kinase binding protein 1	0.92	1.41	0.44		h			
SHANK2	cortactin binding protein 1	2.39	3.2	1.58	h				
SIAT9	sialyltransferase 9	0.18	0.1	0.26		h			
SIM2	single-minded homolog 2 (Drosophila)	2.81	2.79	2.83		h		h	h
SIRT7	sirtuin silent mating type information regulation 2 homolog 7 (S. cerevisiae)	1.68	1.26	2.09					h
SLC12A7	solute carrier family 12 (potassium/chloride transporters), member 7	2.1	2.03	2.17	h	h	h	h	h
SLC16A2	solute carrier family 16, member 2	0.3	0.26	0.35				hm	
SLC20A1	solute carrier family 20 (phosphate transporter), member 1	0.45	0.59	0.32				hm	
SLC2A3	solute carrier family 2 (facilitated glucose transporter), member 3	0.97	1.59	0.35			h		
SLC35A1	solute carrier family 35 (CMP-sialic acid transporter), member 1	1.56	1.08	2.04		h			
SLC9A3R1	solute carrier family 9 (sodium/hydrogen exchanger), isoform 3 regulatory factor 1	1.65	0.85	2.44		hm			
SLK	Ste20-related serine/threonine kinase	0.73	0.46	1					h
SLMAP	sarcolemma associated protein	0.51	0.54	0.47	h			h	h
SMARCA3	helicase-like transcription factor	2.74	3.47	2.01	h	h	h	h	h
SMC2L1	SMC2 structural maintenance of chromosomes 2-like 1 (yeast)	1.87	2.12	1.62		h			h
SMP1	small membrane protein 1	0.74	0.5	0.99		h	h	h	h
SMPD1	sphingomyelin phosphodiesterase 1, acid lysosomal (acid sphingomyelinase)	0.53	0.56	0.5				h	h
SMURF2	E3 ubiquitin ligase SMURF2	0.29	0.42	0.17		h			
SNCAIP	synuclein, alpha interacting protein (synphilin)	1.8	1.23	2.37		hm		hm	
SNPH	syntaphilin	0.53	0.49	0.57		hm		hm	
SOX8	SRY (sex determining region Y)-box 8	0.4	0.23	0.58		h			
SOX9	SRY (sex determining region Y)-box 9	0.27	0.41	0.14	h		h		
SPARC	secreted protein, acidic, cysteine-rich (osteonectin)	0.49	0.69	0.29			h		
SPINT2	serine protease inhibitor, Kunitz type, 2	0.35	0.31	0.4	hm	hm		hm	
SPOCK	sparc/osteonectin, cwcv and kazal-like domains proteoglycan (testican)	2.07	1.7	2.44		h			
SPP1	secreted phosphoprotein 1	9.1	0.99	17.22				h	h
SPRR1A	Small proline-rich protein 1A	0.35	0.04	0.67	h	h		h	h
SPRR1B	small proline-rich protein 1B (cornifin)	0.53	0.42	0.65	h	h	h	h	h
**SPRR2A**	**Small proline-rich protein 2A**	1.4	0.39	2.41					h
SPRR3	small proline-rich protein 3	0.87	0.26	1.48	h			h	

SRPRB	Signal recognition particle receptor, B subunit	1.96	2.12	1.79		h	h		
SRPX	sushi-repeat-containing protein, X chromosome	0.11	0.11	0.11		hm	hm	hm	
SSBP1	single-stranded DNA binding protein	0.76	1.04	0.48		h			
STC1	stanniocalcin 1	5.22	9.31	1.13					h
STC2	stanniocalcin 2	1.45	2.24	0.65	h		h		h
STOML1	stomatin (EBP72)-like 1	1.72	0.87	2.56	h				
STX3A	syntaxin 3A	1.35	0.62	2.09				h	
SULT1A4	Sulfotransferase family, cytosolic, 1A, phenol-preferring, member 4	2.07	1.99	2.15	h	h	h	h	
SURF1	surfeit 1	1.99	1.83	2.15		h			
SUV420H1	suppressor of variegation 4-20 homolog 1 (Drosophila)	2.14	2.66	1.62		h			h
SYK	spleen tyrosine kinase	0.66	0.46	0.85	h		h	h	
T1A-2	lung type-I cell membrane-associated glycoprotein	0.37	0.21	0.52		h	h		
TACSTD1	tumor-associated calcium signal transducer 1	6.35	8.27	4.43		h			
TAF9	TAF9 RNA polymerase II	0.72	1	0.45	h	h	h		h
TAL1	transglutaminase 2	1.42	2.13	0.72					h
**TAPBP**	**TAP binding protein (tapasin)**	1.78	1.41	2.14	hm	hm	hm	hm	hm
TBC1D1	TBC1 (tre-2/USP6, BUB2, cdc16) domain family, member 1	0.65	0.85	0.44	h	h	h	h	h
TBX5	T-box 5	2	2.33	1.67	h		hm	h	h
TCF2	transcription factor 2, hepatic; LF-B3; variant hepatic nuclear factor	0.51	0.49	0.53			hm	hm	
TCF4	transcription factor 4	0.39	0.36	0.42					h
TDGF1	teratocarcinoma-derived growth factor 1	0.41	0.2	0.62		h		h	h
TDRD7	Tudor domain containing 7	1.59	1.06	2.11		h		h	h
TFAP2A	transcription factor AP-2 alpha (activating enhancer binding protein 2 alpha)	0.59	0.68	0.49				h	h
**TFPI2**	**tissue factor pathway inhibitor 2**	0.35	0.35	0.35	h		h	h	h
TGDS	dTDP-D-glucose 4,6-dehydratase	3.04	4.29	1.8	h				hm
TGFB2	transforming growth factor, beta 2	0.53	0.57	0.5			hm	hm	hm
TGFBR2	transforming growth factor, beta receptor II (70-80kD)	0.43	0.48	0.38		hm			
**TGM1**	**transglutaminase 1**	1.53	0.69	2.37		hm			
THAP11	THAP domain containing 11	1.64	1	2.27					h
THBS1	thrombospondin 1	0.14	0.2	0.09	h	h	h		
THBS2	thrombospondin 2	0.1	0.11	0.08		h			
TIMM10	translocase of inner mitochondrial membrane 10 homolog (yeast)	1.71	2.32	1.11	hm		hm	hm	hm
TIMP2	tissue inhibitor of metalloproteinase 2	4.16	4.74	3.58	hm	hm		hm	
TJP2	tight junction protein 2 (zona occludens 2)	0.65	0.97	0.33	hm	h	hm	h	
TK1	thymidine kinase 1, soluble	2.48	2.58	2.37	h			h	
TLE4	BCE-1 protein	0.87	0.28	1.46				h	h
TM4SF1	transmembrane 4 superfamily member 1	0.67	0.47	0.87	hm			hm	hm
TM7SF3	Transmembrane 7 superfamily member 3	0.47	0.49	0.45	h		h	h	
TMEPAI	transmembrane, prostate androgen induced RNA	0.57	0.83	0.3		h	h		

TMOD1	tropomodulin	1.9	1.58	2.21			h	h	
TMOD3	tropomodulin 3 (ubiquitous)	0.38	0.39	0.38	h		hm	hm	hm
**TNC**	**Thrombospondin-1 (TSP-1)**	0.68	0.46	0.9	hm		hm		
TNFAIP2	tumor necrosis factor, alpha-induced protein 2	9.21	11.33	7.09	h	h	h		
TNFRSF1A	tumor necrosis factor receptor superfamily, member 1A	0.64	0.78	0.49	h	h		h	h
TNFRSF6B	tumor necrosis factor receptor superfamily, member 6b, decoy	0.35	0.55	0.15	h	h	h	h	h
**TNFSF10**	**tumor necrosis factor (ligand) superfamily, member 10**	3.52	0.98	6.06					h
TNNI3	troponin I, cardiac	0.57	0.49	0.65	h			h	
TNP1	transition protein 1 (during histone to protamine replacement)	2.03	2.18	1.88	h		h	h	h
TNXB	tenascin XB	15.95	13.79	18.11		h			
TOP2A	topoisomerase (DNA) II alpha (170kD)	2.25	2.24	2.26		h			
TOPBP1	topoisomerase (DNA) II binding protein	2.23	1.97	2.49	hm				h
TP73L	tumor protein 63 kDa with strong homology to p53	0.84	0.34	1.35		h			
TRA1	tumor rejection antigen (gp96) 1	0.53	0.66	0.4		hm	h		
TRIM29	tripartite motif-containing 29	0.75	0.21	1.3				h	h
TROAP	trophinin associated protein (tastin)	1.83	2.13	1.53	h	h	h	h	
TSC1	tuberous sclerosis 1	1.72	1.44	2		h			hm
TSC22	transforming growth factor beta-stimulated protein TSC-22	1.73	2.08	1.39	h	h	h	h	
TSPAN2	tetraspan 2	0.5	0.59	0.41		h			h
TSPAN4	tetraspanin 4	0.61	0.75	0.47	h	hm		h	
TSPAN7	tetraspanin 7	1.86	1.06	2.65	h		h	h	
TTID	titin immunoglobulin domain protein (myotilin)	0.49	0.37	0.6				hm	hm
TU12B1-TY	TU12B1-TY protein	0.49	0.52	0.46					h
TXNIP	thioredoxin interacting protein	0.69	0.3	1.07				h	
UAP1	UDP-N-acteylglucosamine pyrophosphorylase 1	0.33	0.45	0.2	h	h	h	h	h
UBE2H	ubiquitin-conjugating enzyme E2H (UBC8 homolog, yeast)	0.52	0.46	0.58	hm	hm	hm	hm	
UBE2L6	ubiquitin-conjugating enzyme E2L 6	1.82	2.28	1.37	h	h		h	h
UP	uridine phosphorylase	0.74	1.08	0.39	hm		hm	hm	hm
USP6	ubiquitin specific protease 6 (Tre-2 oncogene)	0.53	0.48	0.58	h		h	h	
VAMP3	vesicle-associated membrane protein 3 (cellubrevin)	0.58	0.45	0.71		h			
VASP	vasodilator-stimulated phosphoprotein	0.37	0.35	0.4		h			h
**VAT1**	**vesicle amine transport protein 1**	0.57	0.7	0.44	h				
VDR	vitamin D (1,25- dihydroxyvitamin D3) receptor	0.43	0.37	0.49	h	h	h	h	h
**VEGFC**	**vascular endothelial growth factor C**	0.43	0.52	0.34		h			
VGLL1	Vestigial like 1 (Drosophila)	2.14	3.26	1.02	h	h			
**VIM**	**vimentin**	0.11	0.17	0.05	h	hm	h	h	h
VPS28	VPS28 protein	0.31	0.32	0.31	h		h	h	
VSNL1	visinin-like 1	1.54	0.54	2.53				h	
WFDC2	WAP four-disulfide core domain 2	1.93	2.14	1.72	h	h	h		

WHSC1	Wolf-Hirschhorn syndrome candidate 1	0.61	0.81	0.4			h		
WRB	tryptophan rich basic protein	0.55	0.64	0.46		h			
XG	Xg blood group (pseudoautosomal boundary-divided on the X chromosome)	0.27	0.17	0.37	h	h	h	h	h
YAF2	YY1 associated factor 2	0.47	0.46	0.48				h	h
YAP1	Yes-associated protein 1, 65 kD	2.99	5	0.98	h		h		
ZAK	sterile-alpha motif and leucine zipper containing kinase AZK	0.64	0.87	0.41		h	h	h	
ZNF239	zinc finger protein 239	2.41	3.19	1.63	h				
ZNF266	zinc finger protein 266	1.88	1.24	2.51		h		h	
ZNF336	zinc finger protein 336	0.34	0.27	0.4					h
ZNF42	zinc finger protein 42 (myeloid-specific retinoic acid- responsive)	0.19	0.24	0.14			h		
ZNF589	Zinc finger protein 589	0.51	0.5	0.53		h	h		h

Shown is a list of differentially expressed genes with predicted NF-kB binding motifs in their promoter regions from head and neck squamous cell carcinoma (HNSCC).
[a] refers to the ratios when compare the means of gene expression levels of HKC with 5 UM-SCC cell lines of wt p53-deficient status (ΔW), with 5 UM-SCC with mutant p53 status(ΔM), or with the 10 tumor cell lines (ΔT). [b] refers to 5 NF-κB binding matrices from Transfac: RELA(p65), NFκB1(p50), cRel, NF-κB_Q6_01 and NF-κB_Q6. 'h' indicates predicted regulation in human promoters, 'hm' indicates predicted regulation in both human and mouse promoters.
Gene symbol and description in bold represent known NF-κB target genes from [86-88].
