# Supplementary material for: Systems biology-defined NF-κB regulons, interacting signal pathways and networks are implicated in the malignant phenotype of head and neck cancer cell lines differing in p53 status
Source: Genome Biol. 2008 Mar 11;9(3):R53. doi: 10.1186/gb-2008-9-3-r53 (PMC2397505; doi:10.1186/gb-2008-9-3-r53)
Supplement: Additional data file 2 — Gene Ontology annotations for NF-κB target genes in wild-type p53-deficient, mutant p53 and wild-type plus mutant p53 subsets of UM-SCC cells. [file gb-2008-9-3-r53-S2.pdf]

Supplemental Table S2. Gene Ontology Annotation of NF-κB target genes in HNSCC

Function Name	Number [a]	% [b]	*P* value [c]
wt p53-deficient			
epidermis development	13	2.82	1.29×10^{-7}
cell differentiation	22	4.77	1.80×10^{-4}
angiogenesis	7	1.52	3.67×10^{-3}
blood coagulation	9	1.95	4.22×10^{-3}
cell-cell signaling	19	4.12	1.08×10^{-2}
cell adhesion	25	5.42	2.81×10^{-2}
negative regulation of cell proliferation	12	2.6	3.04×10^{-2}
cell surface receptor linked signal transduction	12	2.6	3.90×10^{-2}
mt p53			
antigen processing, endogenous antigen via MHC class I	6	1.18	1.20×10^{-5}
angiogenesis	9	1.76	2.38×10^{-4}
positive regulation of I-kappaB kinase/NF-kappaB cascade	11	2.16	1.26×10^{-3}
epidermis development	9	1.76	1.08×10^{-3}
response to oxidative stress	6	1.18	7.11×10^{-3}
cell adhesion	30	5.88	9.33×10^{-3}
cell differentiation	20	3.92	1.19×10^{-2}
negative regulation of cell proliferation	14	2.75	1.54×10^{-2}
cell-cell signaling	20	3.92	2.64×10^{-2}
development	27	5.29	3.42×10^{-2}
regulation of progression through cell cycle	20	3.92	3.44×10^{-2}
cell-matrix adhesion	6	1.18	4.46×10^{-2}
wt+mt p53			
epidermis development	11	2.57	7.41×10^{-6}
cell differentiation	21	4.91	3.24×10^{-4}
angiogenesis	8	1.87	4.05×10^{-4}
fatty acid biosynthesis	6	1.4	2.70×10^{-3}
blood coagulation	8	1.87	1.28×10^{-2}
positive regulation of I-kappaB kinase/NF-kappaB cascade	8	1.87	1.99×10^{-2}
cell-cell signaling	17	3.97	2.89×10^{-2}
transmembrane receptor protein tyrosine kinase signaling pathway	7	1.64	2.89×10^{-2}
cell adhesion	24	5.61	2.89×10^{-2}

Putative NF-κB predicted genes in head and neck squamous cell carcinoma (HNSCC) from supplemental Table S1 were analyzed through gene ontology (GO) biological process by using Onto-Express [91].
[a] refer to gene number for an identified functional GO category in this gene set. [b] refer to the percentage of genes in each functional GO category. [c] indicate corrected *P* value for statistically significant enrichment (cutoff $P<0.05$).
