# Supplementary material for: Systems biology-defined NF-κB regulons, interacting signal pathways and networks are implicated in the malignant phenotype of head and neck cancer cell lines differing in p53 status
Source: Genome Biol. 2008 Mar 11;9(3):R53. doi: 10.1186/gb-2008-9-3-r53 (PMC2397505; doi:10.1186/gb-2008-9-3-r53)
Supplement: Additional data file 3 — Network lists generated by IPA based on gene sets regulated by NF-κB subunits RELA/p65 and NFκB1/p50. [file gb-2008-9-3-r53-S3.pdf]

Supplemental Table S3. The networks in NF-κB target genes of HNSCC

Network [a]	Molecules in network [b]	Score [c]	Top functions [d]
RELA target genes in wild type p53-deficient status			
1	Ap1, **BIRC2,** Cbp/p300, **CDKN1A, CSF1, CSF2, ETV1, FASN, HTATIP, IKBKE,** IL1, **IL6, IL8, IL32, IL1A, IL1R2, IRF4, MT2A, NEDD4L, NR4A1, PIM1, PPARBP, PTGES,** ***RELA,*** RNA polymerase II, **RPS6KA1, RPS6KA2, SFN, TFPI2, TNFAIP2, TNNI3, UBE2H, UBE2L6, VDR, YAP1**	52	Cellular Growth and Proliferation, Hematological System Development and Function, Immune and Lymphatic System Development and Function
2	**ACLY, ADFP, AKAP12, ALB,** ALOX5AP, **ALPP,** AMELX, **ARL4C,** CEBPA, CPB2, CPS1, CYP3A4, **DSG3,** ELAVL1, FOS, **GPR109B, ICAM1, IL6, IL8,** KRT5, **KRT14, LCN2, MT1L,** NFYB, NR3C1, PKP1, PRTN3, SCAP, **SERPINB1, SERPINB7, SPINT2, SULT1A3,** THBD, **TM7SF3,** TNFAIP6	25	Dermatological Diseases and Conditions, Gene Expression, Cell Death
3	**BMP4,** CSPG2, CTNNB1, DLL1, **ERCC1,** ERCC5, **FBN2,** FST, **FSTL3, GAA,** HOXA5, IFI16, **INHBA,** INHBB, JAG1, JUNB, **MCAM, MDFI,** MFAP2, **MFAP5, MSX1,** NDN, NOTCH1, **PARP2, PEG3, PHLDA1,** POLB, RPA, **S100A2, SAT1,** SGK, **SPRR1B,** TCF7L2, TERF2, TP53	19	Cellular Development, Connective Tissue Development and Function, Skeletal and Muscular System Development and Function
4	14-3-3, Actin, Akt, CALM2, Calmodulin, **CASP1, CASP4,** CASP5, CASP14, CEACAM1, D830050J10RIK, **DMAP1,** F Actin, **GNAI2, GNB1, ICAM1, ITPKA, KIF23, MARK4, MLPH,** MYO5A, **NLRP1,** NOD1, NOS3, PDK1, PLEC1, PLS1, PLS1, RAB8B, S100A8, **S100A9, SYK, USP6,** VASP, **VIM**	18	Post-Translational Modification, Cellular Movement, Hematological System Development and Function
5	**ABCA1,** AUP1, CAV1, **CIT,** CYR61, DLG4, **FLOT1,** FLOT2, **FN1,** GJB1, **GJB2,** HRG, Integrin α;, **ITGA2, ITGA5,** ITGA8, **ITGB4,** LAMA1, LAMA2, **LAMA3,** LAMA5, LAMB1, LAMB3, LAMB1-2, LAMC1, LAMC2, LAMC3, LIN7A, **PLAT,** PLG, **SERPING1,** TFPI, **THBS1, TIMP2, TNC**	18	Cell-To-Cell Signaling and Interaction, Cellular Movement, Dermatological Diseases and Conditions
6	CBX4, CEBPB, DLEU1, **DUSP6,** E2f, EXOSC1, EXOSC2, EXOSC3, **EXOSC7,** EXOSC8, EXOSC9, FABP4, **FADS2, FADS3,** GJA5, HIST1H4C, Histone h3, **HLTF,** IFI16, **IGF2BP1,** IKZF3, **KRT6B,** Linoleoyl-CoA desaturase, **MYBL2,** MYC, MYLPF, **NUSAP1, RBBP4,** SFRS2, **SFRS11,** SMARCA4, TBX2, **TBX5, TM4SF1,** VLDLR	16	Cell Cycle, Cellular Assembly and Organization, DNA Replication, Recombination, and Repair
7	**ABCG2,** ACACA, ACSL1, **ACSL4, ACSL5,** ARNT2, CFD, **COL12A1, CYBA, DNAJB5,** EPAS1, **FASN,** GADD45G, KLF2, **LAMB3,** LEP, Long-chain-fatty-acid-CoA ligase, **MGLL,** MTTP, **MVD,** NCF1C, NFE2L2, **NMB,** NMBR, NR1H3, PPARG, PPARGC1B, **RAMP2,** RETN, **SCD,** SIM1, **SPRR1A,** SREBF1, UCP1, UGT1A6	16	Lipid Metabolism, Molecular Transport, Small Molecule Biochemistry
8	**AMY2A, ARMET, BTG3,** CDKN2A, **CDSN, CPA1, EGLN1,** EP300, EPAS1, FEN1, FOXA2, FOXA3, GCG, HIF1A, **HOXB9,** IGFBP2, IGFBP3, **IL6, KIF2C, KLK5,** KLKB1, KPNB1, **P8, POU3F4,** PTF1A, **RAD1,** RAD17, RBPSUHL, RECC5, RFC3, RFC4, **SOX9,** SUZ12, TCF7L2, TWIST1	16	Gene Expression, Hematological Disease, Respiratory Disease
9	**ARID3A, ASS1,** AURKB, CCNE1, CD99, CDC25B, **CDKN1A, CENPA,** CENPB, **COL7A1,** Cyclin E, DLGAP1, **EFNB1,** EFNB2, ENG, Ephb, EPHB1, **EPHB2, FEZ1, FOXM1,** GRIA2, GRIN1, GRIP1, **KLF6,** PARP1, PICK1, PLA2G4A, PLAU, PRKCE, PRKCZ, **SHANK2,** SLC9A3, SP1, TEAD1, **VGLL1**	14	Nervous System Development and Function, Cellular Assembly and Organization, Cell Cycle
10	BAZ1A, **BOK,** CHRAC1, CPS1, **DUSP10, DUSP22,** E2F1, FGFR3, **FYB,** ITK, Jnk, **KRT8,** LCK, **LMO7,** MAP2K7, MAP3K2, MAP3K5, MAPK8, MAPK10, **POLE3,** PRKCE, PTK2B, **RAD54L,** RAF1, **RSU1, SAPS3, SH2D2A,** SKAP1, SKAP2, SMARCA5, **TK1,** TP53BP2, **VPS28,** YWHAE, YWHAG	14	Cell Signaling, Cellular Assembly and Organization, Amino Acid Metabolism
11	BYSL, CD81, CDH1, Erm, ETS2, FOS, GLI1, IGF1, IGF2, **IGFBP6,** JUN, **KRT8,** KRT18, KRT19, MSN, **PALLD, PTGFRN,** PTH, PTHLH, PTHR1, **PTHR2, TROAP, TSC22D1,** VIL2	7	Skeletal and Muscular System Development and Function, Connective Tissue Development and Function, Gene Expression
RELA target genes in mutant p53 status			

1	**ALPP,** Ap1, **ARL4C, ARTS-1,** Cbp/p300, **CDKN1A, CEBPA, CSF2, CYP3A4, FABP4, GPR109B,** Histone h3, IL1, **IL6, IL32,** IL1/IL6/TNF, **IL1A, IL1B,** Il1r, **IL1R2, IL2RA, KLF6, MT2A, MUC1, NR4A1, PLK3, PPARBP, PTGES, REL,** ***RELA,*** **SAA1, TFPI2, TNFAIP2, TOPBP1, VDR**	46	Immune and Lymphatic System Development and Function, Tissue Morphology, Dermatological Diseases and Conditions
2	ACTB, **ARMET,** BMP7, CASP4, Cbp/p300, **CFB, COTL1, DUSP5, DUSP10, EGLN1,** ENG, **FSTL3,** GBP1, **HIF1A, HOXA13,** IGF2, **INHBA,** INHBB, **MCAM,** MYCN, NOS3, NOS2A, **PARP2, PHLDA1,** PLAUR, POLB, **PTGS1,** PTK2, **SAT1, SERPING1,** STAT1, **TAF9,** TAF4B, TP53, VEGFA	19	Cardiovascular System Development and Function, Organismal Development, Cancer
3	**ABCA1, AKNA,** APOA1, ATF2, **B4GALT5,** CD40, **CD99, CDKN2C,** CTGF, E2f, EGR1, **ERCC1,** ERCC5, ERCC4, FASLG, **FLOT1, FN1,** FOS, **GTF2H2, MT2A, MYBL2,** NR2F1, **NR4A1, NUSAP1,** PLAU, PLAUR, POMC, RBL1, **SERPINB7,** SP1, TFIIH, TNF, **TNFRSF6B,** TNFSF14, **VIM**	19	Cell Cycle, Cell Death, Cellular Growth and Proliferation
4	Aldehyde dehydrogenase (NAD), Aldehyde dehydrogenase (NADP), ALDH, **ALDH1A2, ALDH1A3, ALDH3B2,** ALOX5AP, **BMP2K,** Cbp/p300, CEBPB, CPB2, CRSP2, **CYP3A4, DAZAP2,** DCAMKL1, **ETS2, HLA-C,** IGF1, **KRT6B,** MBP, MMP1, MMP3, MMP13, **MT1L,** NR3C1, **PSG1,** PTH, PTHLH, **PTHR2,** QKI, **RBPMS,** SPP1, **TMEM176A,** TNF, ZFYVE9	16	Gene Expression, Connective Tissue Development and Function, Skeletal and Muscular System Development and Function
5	CTGF, **CYR61,** DCN, FBLN1, FGA, **FN1,** HOXD3, IGF1, Igfbp, **IGFBP2,** IGFBP5, **IGFBP6,** Integrin α, **ITGA2,** ITGA4, **ITGA5,** ITGA8, **ITGB4,** ITGB6, ITGB8, JRK, **LTBP2,** MIA, MMP7, MMP11, **MT1E,** MTPN, PLAT, **PVALB,** SDC1, **SLMAP,** TGM2, **THBS1, TIMP2, TSPAN7**	16	Cellular Movement, Cancer, Cell-To-Cell Signaling and Interaction
6	ALB, **AMY2A, CEBPA,** CFD, CYP2C40, **DARC, EPHX1, ESPL1,** F8, **FBN2,** FOSB, FOXA1, FOXA2, FOXA3, **GAA,** HOXA5, IL8, IVL, JAG1, **LCN2, MFAP5,** MTPN, NFYB, NOTCH1, **OAT,** PTTG1, RGD:632285, RUNX2, SLC2A4, **SPINT2,** SPP1, **SULT1A3, TM7SF3,** TNFAIP6, VWF	14	Cellular Growth and Proliferation, Cellular Development, Embryonic Development
7	**ADFP,** ATF2, Cbp/p300, CGN, CLDN1, CLDN5, **DNAJB5, EIF2AK3,** GADD45G, **H1F0,** HBP1, HMGB1, **HOXD9, HSPB8, MAGEF1, MGLL,** MIA, MYOD1, NCOA1, NFE2L2, **NR4A1, PDK2,** PPARG, PPARGC1A, RB1, RUNX2, RXRB, SAFB, **SCD, SOX9,** TJP1, **TJP2,** TNF, VEGFA, VNN1	14	Gene Expression, Cell Morphology, Cellular Function and Maintenance
8	ATF2, ATP2A2, BCL2, **C11ORF9,** CASP9, DOK2, **DUSP6,** DUSP22, **EFNB1, ETV1,** FES, **FYB,** IL13RA1, **IL4R,** ITK, LCK, Mapk, MAPK1, MAPK8, PLA2G4A, PLAG1, **PLCG2,** PTK2, **PTPN7,** PTPRH, **S100A2,** SERCA, SH2D2A, SIRPA, SKAP1, STAT3, **TNFRSF1A, TSPAN4,** VEGFA, WAS	14	Cell Death, Hematological Disease, Immunological Disease
9	ARHGDIA, ARHGDIB, ARHGDIG, ARNT2, **CCL16,** CCR1, **CD52,** CDC42, **CDC42EP2, CIT,** COL12A1, **CYBA,** DIAPH1, DIAPH3, **GCA,** LAMA2, **LAMA3, LAMB3,** LAMB1-2, LAMC3, LCP1, MCF2, NCF4, PKN1, **RAC2,** Rac/Cdc42, RGNEF, Rho, **RHOA, RHOC,** RHPN1, RND3, Rock, ROCK2, RTKN	13	Cell Signaling, Cellular Assembly and Organization, Cell-To-Cell Signaling and Interaction
10	**ACLY, ACSL4,** Akt, APOA1, **BRMS1,** CABIN1, CALM2, Calmodulin, CCNB2, CENPA, CYB5A, E2F4, ESR1, **FADS2, FOXM1, GCG,** HDAC1, HIST2H2AA3, **HIST2H2BE, HLTF,** LRCH4, LTF, **MVD,** NCOR2, NFATC2, NOS3, PCNA, **PEA15,** PML, **POU3F4,** RBL1, **RBM38,** SREBF1, TNF, VEGFA	13	Gene Expression, Lipid Metabolism, Small Molecule Biochemistry
11	14-3-3, Actin, ACTN, **ACTN3, AP3B1,** AP3B2, CAPN8, CD44, CEACAM1, DES, G alpha, **GNA15, GNAI2,** GPSM1, GSN, ITGB3, **ITPKA,** MSN, **NME4,** NOS3, **PALLD,** PDK1, PLEC1, PLS1, **POLR2L,** PTK2, RAB8B, RNA polymerase II, **SFN,** SYNPO2, TTC1, **UPP1,** VIL2, **VIM,** YWHAE	11	Cellular Assembly and Organization, Cellular Movement, Cell Morphology
12	14-3-3, Akt, AKT1, **BOK,** CASP9, **CNKSR1,** CPS1, D830050J10RIK, E2F1, **FEZ1, GBAS, LMO7,** MAP2K5, MAP3K5, MCL1, NIPSNAP1, PPM2C, PPP1R12A, PPP2CA, PRKCD, PRKCE, PRKCI, PRKD1, RAF1, RB1, RBL1, **RSU1, TBC1D1, TK1, VIM, VPS28,** YWHAB, YWHAE, YWHAG, YWHAZ	11	Cancer, Cell Death, Respiratory Disease
13	B2M, CALR, CANX, CIITA, FCGRT, **GJA5,** H2-LD, HLA-A, **HLA-DMA, HLA-DMB,** HLA-DOB, HLA-E, HLA-F, LPL, MHC Class I, NFKB1, **PDIA2,** PDIA3, RFX5, Tap, TAP1, TAP2, **TAPBP**	4	Immune Response, Protein Trafficking, Nervous System Development and Function

NFkB1 target genes in wild type p53-deficient status

1	**ABCG2, ADFP,** Ap1, Cbp/p300, **CDKN1A, CSF2, FDPS, FOXM1, GSTM2, HR, IVL,** Jnk, **JUND, KCNN4, KLF6, KRT8, LAMB3, LAMC2, MGLL, MSX1, MT2A,** *NFKB1,* **NR4A1, PIM1, PPARBP, PPARG, PRNP, PTGES, PTGS2, SAT1, SCD, SPRR1A, SPRR1B, TP73L, VDR**	53	Dermatological Diseases and Conditions, Cellular Development, Hair and Skin Development and Function
2	ABCB1B, **ACAA2,** ACACA, **ACSL5, AOX1,** ARNT2, CDK2, **CDK2AP1, CGN,** CYB5A, **CYBA,** CYBB, **DUSP4,** EGR1, ELOVL6, FABP5, **FADS2, FDPS, FEZ1, GJB2,** HSD17B1, **IL32,** INSIG1, MVD, OCLN, PRKCZ, **PTGES,** RELA, **SCD, SIM2,** SREBF1, SREBF2, TJP2, **TNFAIP2, UBE2H**	22	Lipid Metabolism, Molecular Transport, Small Molecule Biochemistry
3	**AGER,** Alcohol group acceptor phosphotransferase, APAF1, CASP2, **CASP4,** CASP9, CDK8, CSNK1D, **CTSH, DUSP22,** ETS1, FHL2, GSTP1, HMGB1, HSPB1, MAP2K7, MAPK1, **MCAM,** MYB, NEK2, **NLRP1, NLRP2, PHLDA1,** PLK3, PRKCD, **PRKX, PTPRA, PTPRJ,** PYCARD, **RASD1, S100A2, S100A4, THBS2, TOP2A,** TP53	19	Cancer, Cell Death, Gastrointestinal Disease
4	A2M, ADAM17, **ALB, APBB2,** APLP2, APP, **ARL4C,** CCND3, CEBPA, CEBPD, CFD, **CKAP4, CXCL14, EPPB9,** FGF2, FOSB, **GAA, GAL,** GAPDH, GATA2, GFI1, HES1, HMOX1, **ICAM1,** IL8, **IVL,** KLF2, **MALT1,** NFATC1, NOTCH1, **OAS2,** SDF-1, **SPINT2, SULT1A3,** TNFAIP6	17	Cellular Growth and Proliferation, Gene Expression, Tissue Morphology
5	ADCY2, **DDEF1, DEAF1, EHD1, EPHB2,** FURIN, G protein beta gamma, GNAI2, **GNB1,** GNB2, GNB4, GNG11, **GNG12, GPC1,** HRAS, **IGF2,** IGF1R, IGFALS, **IGFBP6, LIPG,** MATK, **MT1L,** NCF2, NR3C1, PCSK5, **PTK6,** PXN, RAF1, **RARRES3, RSU1,** SH2D3C, SNAP29, SRC, **TDGF1,** TTC1	17	Cell-To-Cell Signaling and Interaction, Cellular Assembly and Organization, Cell Signaling
6	**BCAT1, BOK,** CANX, CEBPE, Cyclin D, E2F3, **FADS2, FADS3, FBN2, FOXM1, FSTL3,** GCK, GFI1, ID1, IFI16, **IL6,** INHBA, Linoleoyl-CoA desaturase, MDM4, MYC, **NEUROD2,** NEUROG3, **PMP22,** RB1, **RHOB,** ROCK1, ROCK2, SERPINE1, SMAD3, **SMURF2, SUV420H1,** TCF7L2, **TGFBR2,** TGM2, ZFP36	15	Gene Expression, Cellular Growth and Proliferation, Cell Cycle
7	ADAM9, **ANPEP,** AUP1, CD9, CD46, CD63, CD81, **CD151, CRIP2,** CYR61, **FN1, GULP1,** HOXD3, Integrinα, ITGA1, **ITGA2,** ITGA3, ITGA4, **ITGA5, ITGA6,** ITGA8, **ITGB4, LILRB3,** LRP1, MIA, MMP16, PLAG1, **PLCD1,** PLEC1, **PTGFRN,** TGM1, TGM2, **THBS1, TIMP2,** TSPAN4	15	Tissue Development, Cellular Movement, Cell-To-Cell Signaling and Interaction
8	**AARS,** AATF, ACSL1, Aldehyde dehydrogenase (NAD), ALDH, **ALDH1A3,** ALDH1A7, ALDH3A1, **ALDH4A1, ALDH5A1,** ALDH9A1, **ASS1,** ATP5B, **BCAP29,** BCAP31, **CHKA, CKB,** CLIC4, ESRRA, **HLTF,** HUS1, IGH-5, **MDK, MPHOSPH6,** NCL, **RAD1,** RAD17, RECC5, RFC4, SMARCA4, **SNPH,** SP1, STX1A, **VAMP3,** YWHAZ	15	DNA Replication, Recombination, and Repair, Lipid Metabolism, Small Molecule Biochemistry
9	14-3-3, Actin, ACTN, **CALD1,** Calmodulin, CAMK2B, CEACAM1, D830050J10RIK, **DMAP1, EPB41,** F Actin, GCG, **GHRH, ICAM1,** IQGAP1, **ITPKA,** MAP2, MARK4, **MLPH,** MYO5A, PDK1, PFN, PLEC1, PLS1, PLS1, POU3F4, **PTPRA,** RAB8B, TES, **TOP2A,** TPM1, Tropomyosin, **VASP, VIM,** YEATS4	14	Cellular Assembly and Organization, Cell Morphology, Endocrine System Development and Function
10	**ABCA1,** ABCB1, ATP2A3, **BMP4,** BYSL, **COL7A1,** DLG4, ETS1, ETV4, F3, F7, F8, F10, **F8A1,** FLI1, **FLOT1, FST,** GRIA2, HGF, **KCND2, KRT8, KRT16,** KRT18, KRT19, LIN7A, LRP2, **MUC4,** MYH7, **PRG1,** SP1, TEAD1, TNFSF14, **TROAP,** USF1, **VGLL1**	14	Hematological Disease, Gene Expression, Organismal Survival
11	**ARMET,** BIRC5, **CA9, CA12,** Carbonate dehydratase, Cbp/p300, **CDSN,** CITED2, COPS5, CREBBP, EPAS1, F2, F11, **F2RL1,** Fibrin, HIF1A, **HOXB9, IGF2,** IGFBP2, IGFBP3, IGFBP5, **KLK5,** KLKB1, **PGF,** PLAU, PLG, **PPARBP,** PROC, SELE, **SERPINE2, SERPING1, TP73L,** Vegf, VEGFA, WT1	14	Organismal Injury and Abnormalities, Hematological Disease, Cancer
12	AEBP2, **CD59, CEP250,** CHAF1A, E2f, EZH2, FOXC1, FOXC2, G alpha, **GNA15,** HIST1H4C, Histone h3, **HLTF,** HOXA11, **IL6,** MAD1L1, **MBD1,** MEIS1, MGMT, **MLL,** MLL2, MTA2, **MYBL2,** NCAPD2, NEK2, PELP1, **PRAME, RBBP4,** RNA polymerase II, **RPS6KA2,** SET, SETDB1, **SMC2,** SMC4, TP53BP2	12	Cellular Growth and Proliferation, Hematological System Development and Function, Immune Response
13	**AKAP12, ALPP,** APC/APC2, ATF4, AXIN1, AXIN2, BIRC2, **BNC1,** CLU, CTNNB1, CYP19A1, DVL3, **FHL1,** FHL2, **FLRT2,** GLI1, Gsk3, HIVEP3, HNRPA1, JUN, MACF1, MACF1, **MDFI,** NFYB, **PPP2R5B,** PTH, PTHLH, **PTHR2, RNPS1,** RUNX2, SAP18, **SOX8,** TPM1, **TSC22D1, UBE2L6**	12	Cellular Development, Connective Tissue Development and Function, Tissue Development

14	**CPA1, CTRB1,** PTF1A, RBPSUHL	3	Amino Acid Metabolism, Cellular Development, Small Molecule Biochemistry
NF κB1 target genes in mutant p53 status			
1	**ANPEP,** Ap1, **ATF3, BMI1,** Cbp/p300, **CCND1, CDKN1A, CDKN2C,** Creb, **CSF2,** Ets, **ETS1, FOXM1, GCG,** Histone h3, **HLA-DMB, IL6, IL32, IL1B, IL2RA, IL6ST, KLF6, KLF7, KRT4, MLL, MT2A, *NFKB1,* NR4A1, PLK3, POU3F4, PRG1, PTGS2, REL,** Stat, **TNFAIP2**	42	Cellular Development, Cellular Growth and Proliferation, Tissue Morphology
2	**ABCA1,** Akt, **AKT1, AOX1, ARTS-1, AXIN1, BCL10, CALD1,** Calmodulin, **CYP1A2, CYP3A4, DDEF1, EFS,** F Actin, **FLOT1, GHRH, HR, IL4R, IRS1,** Jnk, **MALT1,** N-cor, **NFE2L2, PEA15, POLR2L, PPARBP, PRNP, PTPRA, PTPRN2, RASA1,** RNA polymerase II, Sos, **TNFRSF1A, TSC1, VDR**	40	Respiratory System Development and Function, Small Molecule Biochemistry, Tissue Morphology
3	**CD151,** CTGF, DCN, **FBLN1,** FGA, **FN1,** IGF1, **IGF2,** Igfbp, **IGFBP2,** IGFBP5, **IGFBP6,** Integrin α, **ITGA2, ITGA5,** ITGA8, ITGAV, **ITGB4,** ITGB5, ITGB6, ITGB8, LRG1, **LTBP2,** MIA, MMP11, **PGF, PLCD1,** Tgf β, **TGFBR2, TGM1,** TGM2, **THBS1, TIMP2,** Vegf, **VEGFC**	18	Cancer, Tumor Morphology, Cellular Movement
4	**ALPP,** ASC2, ATF2, **ATF3, BATF,** EGR1, **GABARAPL1,** GTF2H4, **H1F0,** IGF1, **ITGB4,** IVL, JUN, JUND, MDM2, MEF2C, **MT1G,** NCOR2, **NKX3-1, NR4A1, OGFR,** OPRK1, PENK, **POMC,** PRKCD, PRKD1, **PTGES, RASD1,** RB1, RUNX2, **SLC9A3R1,** SRF, TNF, **VIM, ZAK**	17	Gene Expression, Cellular Growth and Proliferation, Cancer
5	ATP1A1, **ATP1B3,** BCL2, CDC25C, CSNK2B, **CTSH, FXYD3,** HIF1A, HRAS, **MAP4,** MATN1, **MATN2,** MATN4, **MCAM,** MDM2, **MLL,** MMP2, MYCN, Na^+/K^+ exchanging ATPase, NOTCH1, **PHLDA1, PRODH, PTGS1, PTGS2, RARRES3, SNCAIP,** TADA3L, TAF1, TAF5, **TAF9, THBS2,** TP53, TSG101, Ubiquitin, WDR5	15	Cancer, Cardiovascular System Development and Function, Tumor Morphology
6	APOA1, **ARMET, BOK,** CD44, E2F1, **ESPL1,** FGF2, **FLRT2, GTF2H2,** HBB, **HBE1,** HDAC1, HIF1A, **HLTF, HOXA13, ICK, IGF2,** IGFBP5, KLF3, LOX, **LOXL2, MT1E,** NFYB, **NR4A1, PFKP,** PLAU, PLAUR, PTTG1, **RNPS1,** SMARCA4, SNAI1, SP1, Vegf, VEGFA, VHL	15	Cancer, Tumor Morphology, Cellular Movement
7	**ABLIM1,** Akt, **AKT1,** ARFIP2, ARHGDIA, ARHGDIB, ARHGEF2, CASP3, CDC42, **CDC42EP2, CYBA,** CYBB, **DGKG,** ERC1, **FEZ1, FNTA,** IKBKB, **IRF5,** NCF4, NCF1C, **NLRP2,** PAK1, PCTK2, PDK1, **PPFIA1,** PRKCZ, RAC1, **RAC2,** Rac/Cdc42, RAF1, **RSU1, TBC1D1, TDRD7, VIM,** YWHAG	15	Cell Signaling, Free Radical Scavenging, Nucleic Acid Metabolism
8	Aldehyde dehydrogenase (NADP), ALDH2, **ALDH1A3,** ALDH3A1, **ALDH3B2,** APOC2, ARNT2, ATF2, Cbp/p300, **CEACAM5,** CREBBP, **CRIP1, ELL2,** F7, **F8A1,** GCG, **GDF5,** HNF4A, **LRP8,** MAPK9, NCOA, NCOA2, NFATC2, **NP,** NR2F1, NR2F2, **PDK2,** PPARGC1A, **PTGS2, RGS2, SCD, SIM2,** SOX9, TNF, UGT1A1	15	Gene Expression, Small Molecule Biochemistry, Lipid Metabolism
9	A2M, Ap1, **ARL4C, CA2, CA12,** Carbonate dehydratase, CEBPA, **CKAP4,** COL1A1, **CXCL14, DARC,** ELF4, **EPHX1,** FOXA2, **GAL,** GATA1, **GATA2,** GATA3, GFI1, IL8, KLF2, LDB1, **LMO2,** NFATC1, NFATC2, NOTCH1, **OAS2,** PPARG, RB1, SDF-1, **SERPINF1, SPINT2, SULT1A3,** TAL1, TNFAIP6	14	Gene Expression, Cellular Development, Hematological System Development and Function
10	ABCB1, **ATP2A2,** BCL2, **BRMS1, CCND1,** CCND2, CCNE2, CDK2, **CDK2AP1, CDKN1C,** CDKN2A, CDKN2D, CHAF1A, **CHKA, CKB, DUSP4,** EGR1, ESRRA, FGF2, FOSL1, **FST,** LRCH4, **MBD1, MDK,** MDM2, NCL, PCNA, PLN, PML, POLA1, **RBM38, RREB1,** SKP2, **TMEPAI,** TNF	14	Cell Cycle, Cellular Growth and Proliferation, Cancer
11	**B4GALT1, BCAP29,** BCAP31, BCL2, **C11ORF9,** CALR, CD8A, CD8B, **CRIP2,** ETV4, H2-LD, **HLA-C, HLA-E, HLA-F, HSP90B1,** IGH-5, ITGA3, KIR3DL1, LILRB1, LILRB2, LRP1B, MHC Class I, MHC I-α;, **PDIA2,** PLAG1, PLEC1, **S100A2, SACS,** TAP2, **TAPBP,** TSPAN, **TSPAN2, TSPAN4,** TYR, VCL	14	Immune Response, Cell-To-Cell Signaling and Interaction, Hematological System Development and Function
12	**ADFP, BNC1,** Cbp/p300, E2f, EGFR, **FDPS,** GLI1, IGF1, IL1, IL1R2, **IPO11,** KPNB1, LAMA5, **LAMB3,** LAMB1-2, **LAMC2,** MGLL, MMP14, MT1L, **MYBL2,** NCOA2, NR2F2, NR3C1, **NR4A1, POMC,** PPARG, PTHLH, **PTHR2,** RAN, SDPR, **SH3KBP1,** TNC, TNF, VEGFA, WAP	12	Cellular Function and Maintenance, Hematological System Development and Function, Immune and Lymphatic System Development and Function

13	CD44, **CFB,** DOCK1, **ERBB3,** F2, F11, **F2RL1,** FASLG, **FBN2, FBXO2, FN1,** FURIN, **GAA,** ITGAV, **LIPG,** MFAP5, MYC, NOTCH1, **NR4A1,** PAK2, PLAU, **PLCG2,** PLG, PROC, PTK2, RUNX2, **SERPINE2, SERPING1,** SIRPA, SRC, STAT1, TGFB1, **TNFRSF6B,** VEGFA, VLDLR	12	Cellular Movement, Cellular Growth and Proliferation, Cell-To-Cell Signaling and Interaction
14	**ANK1,** Cbp/p300, COL7A1, CTGF, CYB5A, **CYP26A1,** Dctn1-Dync1I2-Hap1-Hd, DLG4, **EREG, FADS2, FHL1,** FHL2, FHL3, **FSTL3, GPX4, HAP1,** Hap1-Hd, HD, ITGB5, **KCND2,** MAPK1, MMP1, **MVD,** NEUROD1, NR2F1, PLA2G4A, **PTPN7,** SKIL, SMAD3, **SMURF2,** SP1, SREBF1, TGFB1, TGM2, VEGFA	11	Gene Expression, Dermatological Diseases and Conditions, Cardiovascular System Development and Function
15	14-3-3, **AARS,** Actin, ACTN, **ACTN3,** ALS2CR2, BCAR1, **BCAR3, CAB39,** CAPN8, CEACAM1, CLIC4, DES, **DNM1, HIST2H2BE, ITPKA,** LMNA, MICAL1, **NME4,** PDK1, PFN2, PLEC1, PLS1, PTK2, RAB8B, **SSBP1,** STK11, TES, **TOP2A, VASP,** VCL, **VIM,** YWHAB, YWHAZ, ZYX	11	Cellular Movement, Connective Tissue Development and Function, Cellular Assembly and Organization
16	Akt, CD59, CEBPB, CGN, CLDN1, CLDN5, **DCAMKL1,** DCN, EGR2, **EHD1,** EPB41, G alpha, **GFRA3,** GNA12, **GNA15,** GNAI2, **GNB4,** GNG7, GNG11, **GNG12,** IGF1, IGF1R, MBP, MMP1, MPZ, **PDE7A, PMP22,** RET, **SAT1,** SNAP29, SRF, TJP1, **TJP2, TMEM176A,** TTC1	11	Nervous System Development and Function, Genetic Disorder, Neurological Disease

Shown are the networks identified by Ingenuity Pathways Analysis software (IPA) 5.0. ‘Regulon’ represents a set of predicted NF-κB target genes predicted by Clustering Of Gene Regulons using Integrated Modeling (COGRIM) in head and neck squamous cell carcinoma (HNSCC). [a] Ordered number of networks was assigned by IPA and sorted by score. [b] Molecules in bold are the predicted NF-κB target genes with 2 fold changes. Genes in red refer to up-regulated, in green down-regulated ones. Molecules in red or green but not in bold are the NF-κB target genes with 2 fold changes identified in other regulons. NFκB1and RELA in italic indicate override genes. Other molecules were either not on the expression array or fold change below 2 fold change. [c] Score > 3 was significant (P < 0.001). [d] List top three functions annotated by IPA.
